# Tautomeric photoswitches: anion-assisted azo/azine-to-hydrazone photochromism†

**DOI:** 10.1039/c9ra02906k

**Published:** 2019-05-21

**Authors:** Juraj Filo, Pavol Tisovský, Klaudia Csicsai, Jana Donovalová, Martin Gáplovský, Anton Gáplovský, Marek Cigáň

**Affiliations:** Institute of Chemistry, Faculty of Natural Sciences, Comenius University Ilkovičova 6 SK-842 15 Bratislava Slovakia marek.cigan@uniba.sk; Department of Pharmaceutical Chemistry, Faculty of Pharmacy, Comenius University Odbojárov 10 SK-832 32 Bratislava Slovakia

## Abstract

The photoswitching properties of two easily synthesized isatin 4-nitrophenylhydrazones were investigated. Although the parent isatin 4-nitrophenylhydrazones exhibit low addressability which hampers their photochromic applications, the addition of strongly basic anions to phenylhydrazone solutions creates a new Vis–Vis photochromic system with the unusual azo/azine-to-hydrazone photo-tautomerization process as the photoswitching mechanism. To the best of our knowledge, this is the first report related to the anion-assisted azo/azine-to-hydrazone photo-tautomerism.

## Abstract 2

The photoswitching behaviour of isatin 4-nitrophenylhydrazones in presence of anions was investigated.

## Graphical abstract

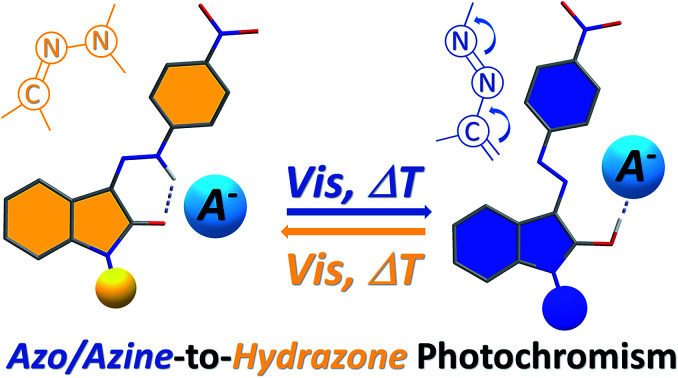

## Introduction

In addition to the traditional performance criteria for photochromic compounds, the ability to isomerize without UV light can be considered a key feature.^1,2^ About two decades ago, several types of all-visible (Vis) light switches based on *E*–*Z* isomerization were reported.^3^

Complementary to these type photoswitches, hydrazones and acylhydrazones found important application in various areas of supramolecular chemistry in the last few years, particularly as molecular switches, but also metallo-assemblies and colorimetric or fluorescent chemosensors.^4–25^ Tautomers are interesting for many reasons, technological as well as fundamental (signaling molecules in sensors, polymer-protecting agents, proton-transfer lasers, information storage devices at a molecular level).^26–31^ Interestingly, capability to establish the azo–hydrazone tautomeric equilibrium led to photochromic oscillators with extremely fast thermal back reaction (T-type photochromic azoderivatives).^32–36^ The azo–hydrazone tautomerization followed by rotation was also proposed as thermal isomerization mechanism in several groups of hydrazone photoswitches. However, to the best of our knowledge, direct photoswitching between azo and hydrazone tautomers was found only in 1-(cyclopropyl)diazo-2-naphthol.^37^ Irradiation of its azo-enol form with a narrowband source in the near-UV range generates different rotameric and tautomeric azo-enol and keto-hydrazone forms that can be interconverted at different irradiation wavelengths.

**Scheme 1:**
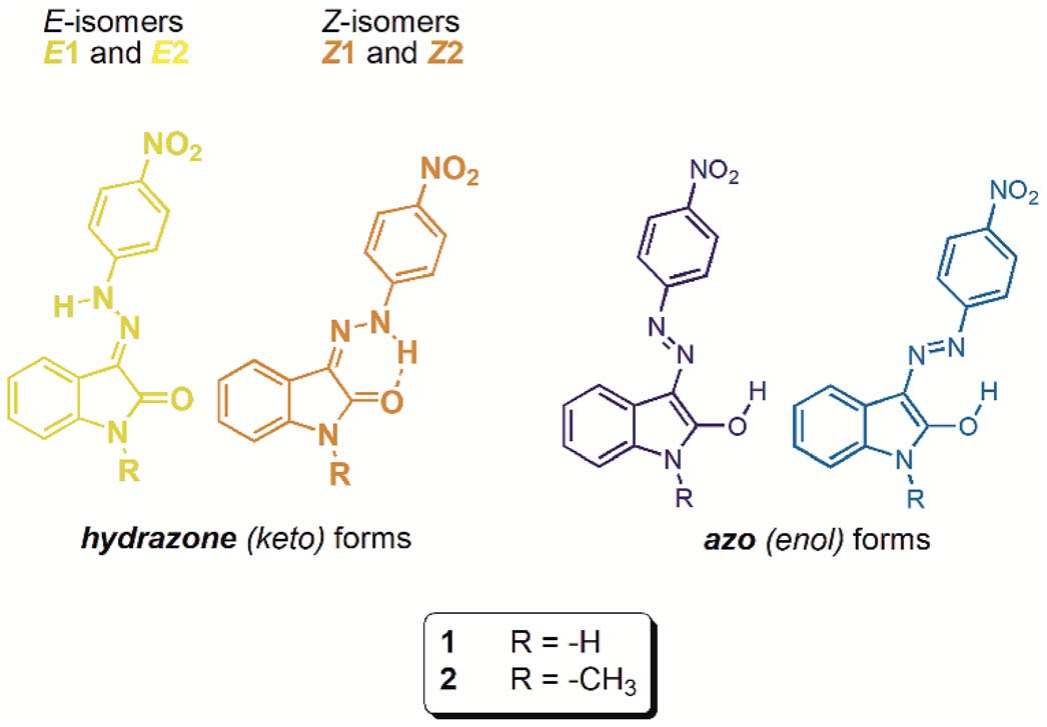
Molecular structure of studied isatin 4-nitrophenylhydrazones 1 and 2.

In this paper, we focused on Vis–Vis photoswitching of two isatin 4-nitrophenylhydrazones (Scheme 1). Although parent isomers exhibit only poor photoswitching (photochromic) behaviour, addition of basic anions creates a new Vis–Vis photochromic system with the unusual azo/azine-to-hydrazone photo-tautomerization process as photoswitching mechanism. Moreover, overall concept could be important in progress of photochromic behaviour of other azo–hydrazone tautomeric switches.

## Results and discussion

### Synthesis

**Scheme 2:**
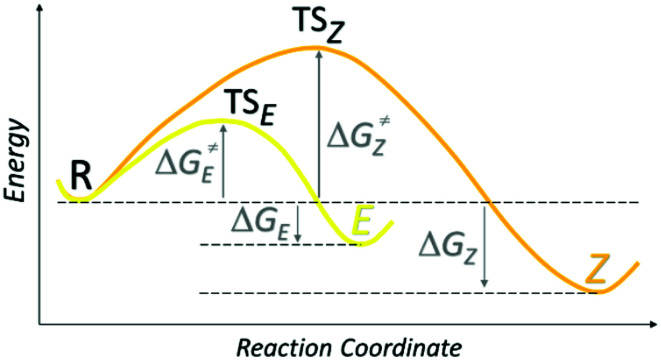
Reaction coordinate (progress of a reaction from reactants to products) of the synthesis of studied isatin 4-nitrophenylhydrazones.

Synthesis of isatin 4-nitrophenylhydrazones (isatin *N*^2^-nitrophenylhydrazones) 1 and 2 represents an educational example of kinetic *vs.* thermodynamic control of a chemical reaction (Scheme 2). Refluxed reaction mixture leads to thermodynamically more stable *Z* isomer (through its stabilization by intramolecular hydrogen bonding – Table 1), whereas stirring of reaction mixture at room temperature affords the corresponding *E* isomer as a kinetic product.

**Table 1:** Calculated relative Gibbs free energies of *E* and *Z* isomers of isatin 4-nitrophenylhydrazones 1 and 2 at the M062x 6-31 + g(dp) level (*T* = 298.15 K; vacuum)

Δ*G*	[kJ mol^−1^]
*E*1	26.8
*Z*1	0
*E*2	28.8
*Z*2	0

Thermodynamically less stable *E* isomers slowly isomerize at room temperature to corresponding more stable *Z* isomers (*τ*_1/2(*E*1)_ = 14.9 h, Δ*G*_*E*1_^‡^ = 99 kJ mol^−1^; *τ*_1/2(*E*2)_ = 6.7 days, Δ*G*_*E*2_^‡^ = 105 kJ mol^−1^; determined in DMSO-*d*_6_), whereas *Z* isomers are stable at room temperature (ESI Fig. S1†).

### Spectral characteristics

*Z* isomers *Z*1 and *Z*2 exhibit typical feature of isatin *N*^2^-phenylhydrazones’ light absorption: the dominant strong Vis light absorption in the blue region (∼430 nm) related to π → π* electronic transition of their yellow hydrazone (keto) form, combined with the indication of weaker absorption in the yellow/orange region (∼590 nm) due to π → π* absorption of their blue azo (enol) tautomeric form in highly polar solvents (Fig. S2†). As expected, the relatively strong intramolecular hydrogen bonding in the *Z* isomers reflects in red-shifted absorption maximum of the *Z* hydrazone form (*λ*_*Z*1_ = 432 nm, *λ*_*Z*2_ = 425 nm) compared to the corresponding *E* hydrazone form (*λ*_*E*1_ = 420 nm, *λ*_*E*2_ = 403 nm; all determined in DMF).

The absence of a transient absorption (related to a triplet excited-state or *Z*–*E* photoisomerization) in transient absorption spectra of *Z*1 and *Z*2 and only weak fluorescence of their solutions (*Φ*_F_ < 0.05) indicate that the S_1_ excited state of these isomers deactivates mainly through fast internal conversion related to intramolecular rotation of nitrophenyl ring (Fig. S3†); as it was recently supported by AIE (aggregation-induced emission) of isatin phenylhydrazone BF_2_ complexes.^38^

### Photochromic behaviour

Irradiation of the *Z*1 isomer at 430 nm in both polar and apolar solvents leads to a gradual small hypsochromic shift in the absorption maximum of the yellow hydrazone form (∼5–10 nm) due to photochemical *Z*-to-*E* isomerization around the C=N double bond (Fig. S4†); maintaining typically poor addressability (separation of the absorption maximum between the *Z* and *E* isomer); with 10% and 40% conversion in the PSS in DMSO-*d*_6_ and CDCl_3_, respectively (determined by ^1^H NMR spectroscopy).

The *Z*2 isomer does not undergo significant visible UV-Vis and/or ^1^H NMR spectral change related to the photochemical *Z*-to-*E* isomerization in both polar and apolar solvents (Fig. S5†). This behavior hampers the photochromic application of the both isomers.

**Fig. 1:**
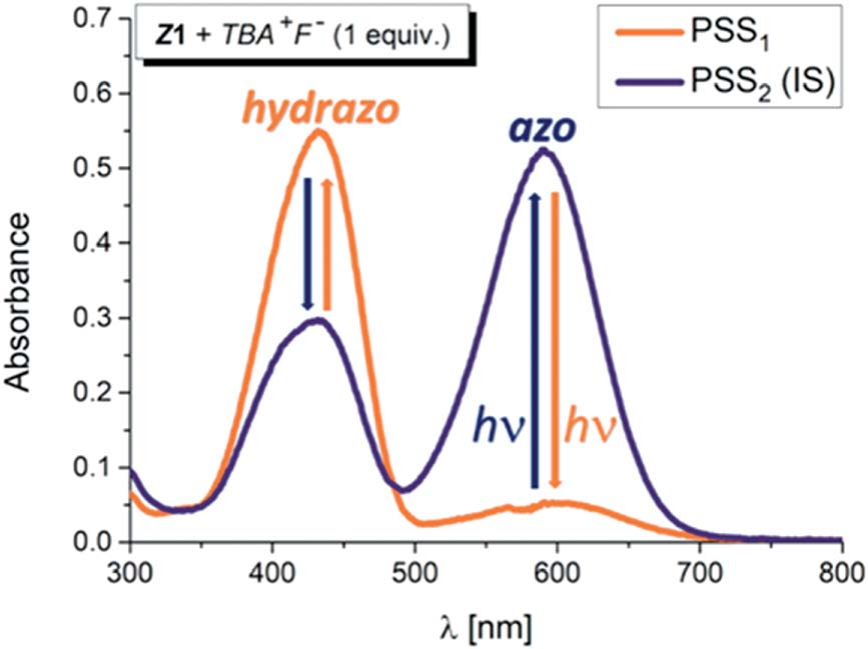
UV-Vis spectral changes during altered irradiation of the *Z*1/TBA^+^F^−^ photochromic system with light of 470 nm and 590 nm wavelength in DMSO-*d*_6_ (initial *Z*-isomer concentration: *c*_*Z*1_ = 10^−4^ M; 0.2 cm cuvette; *T* = 298.15 K; IS – initial state; PSS – photostationary state).

**Fig. 2:**
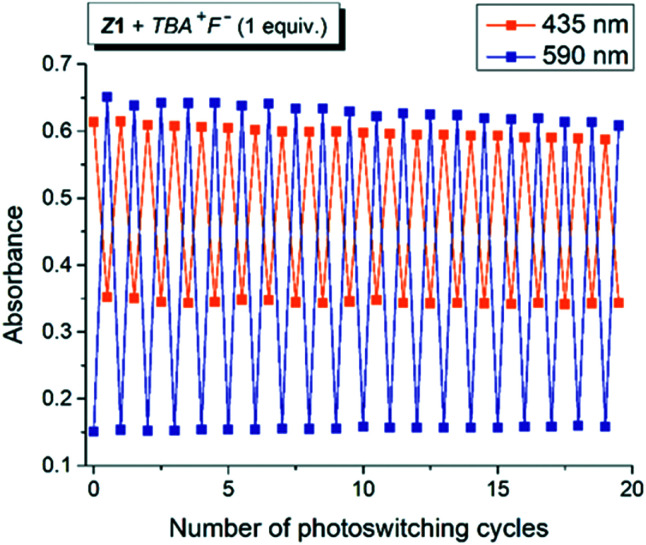
Photoswitching cycles during altered irradiation of the *Z*1/TBA^+^F^−^ photochromic system with light of 470 nm and 590 nm wavelength in DMSO-*d*_6_ (initial *Z*-isomer concentration: *c*_*Z*1_ = 10^−4^ M; 0.2 cm cuvette; *T* = 298.15 K; IS – initial state; PSS – photostationary state).

However, addition of 1 equivalent of strongly basic F^−^ anion to solution of the *Z* isomers in DMSO-*d*_6_ leads to significant increase in the blue azo form absorption at ∼590 nm, accompanied by simultaneous decrease in the yellow hydrazone form absorption at ∼430 nm, and creates a new Vis–Vis photochromic system (Fig. 1, 2, S6 and S7†).

The photochromic efficiency of these systems was studied using UV-Vis and ^1^H NMR spectroscopy. Interestingly, the excitation of the newly-formed azo forms of 1 and 2 with light of 590 nm effectively induces rapid azo-to-hydrazone photoconversion and results in almost complete photoconversion (>95% hydrazone in PSS_1_).

**Fig. 3:**
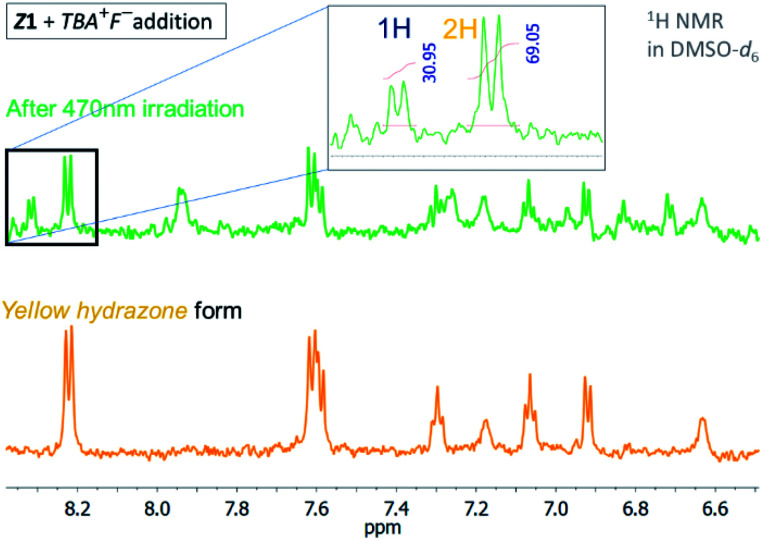
^1^H NMR spectrum of the *Z*1/TBA^+^F^−^ photochromic system in DMSO-*d*_6_ before and after irradiation with light of 470 nm wavelength (*T* = 298.15 K).

**Fig. 4:**
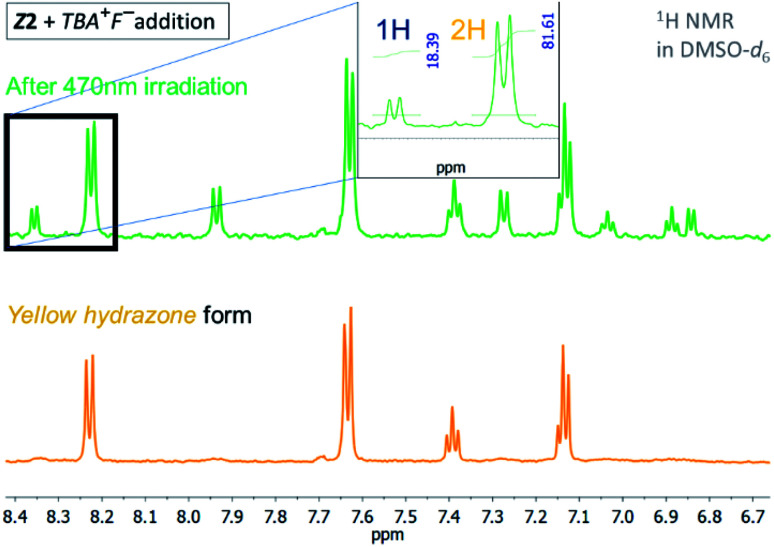
^1^H NMR spectrum of the *Z*2/TBA^+^F^−^ photochromic system in DMSO-*d*_6_ before and after irradiation with light of 470 nm wavelength (*T* = 298.15 K).

The quantum yield of the photoconversion was determined to be 9.5 ± 0.5% for *Z*1/F^−^ and 23.6 ± 1.1% for *Z*2/F^−^ photochromic system, respectively (Table 2). This process is accompanied by a significant solution colour change from blue to yellow. Subsequent irradiation of yellow hydrazone form with light of 470 nm wavelength (or 405 nm) leads to disappearance of its absorption band, while a previous absorption band of blue azo form grows in. Solution colour thus effectively returns to blue. The irradiation yields a PSS_2_ consisting of initial 49% azo form (51% hydrazone form) in the *Z*1/F^−^ and of initial 30% azo form (70% hydrazone form) in the *Z*2/F^−^ photochromic system (Table 2; Fig. 3 and 4).

**Table 2:** Photochemical parameters of the studied photochromic systems *Z*1/F^−^ and *Z*2/F^−^ measured in DMSO-*d*_6_ solution (*T* = 298.15 K)^a^

Photochromic system	Azo–hydrazo PSS (% azo)	Hydrazo–azo PSS (% azo)	*Φ*_A–H_ (%)	*Φ*_H–A_ (%)
*Z*1/F^−^	98	49	9.5 ± 0.5	0.19 ± 0.04
*Z*2/F^−^	96	30	23.6 ± 1.1	0.11 ± 0.02

a *Φ*_A–H_ – photochemical quantum yield of blue azo form transformation to yellow hydrazone form; *Φ*_H–A_ – photochemical quantum yield of yellow hydrazone form transformation to blue azo form.

Although the photoconversion of the both hydrazone forms is far from ideal (complete) and has quite poor efficiency (low photochemical quantum yield; *Φ*_H–A_ – Table 2), the both photochromic systems exhibit excellent addressability and switching amplitude in absorbance (absorbance change). The composition of the PSS_2_ in DMSO-*d*_6_ is thermally not stable and the amount of azo form slightly decreases to 70% of the initial value in the *Z*1/F^−^ photochromic system (*k* ∼1 × 10^−2^ min^−1^; *t*_1/2_–70 min; Fig. S8†). The PSS_1_ composition is approximately 10-fold stable and the amount of azo form slowly increases to its equilibrium value in 760 min (*k* ∼9 × 10^−4^ min^−1^; *t*_1/2_–750 min; Fig. S9†).

For a sustainable application as a molecular photoswitch, a compound must have low photochemical fatigue to allow for large number of switching cycles. The overall process related to the mutual yellow hydrazone and blue azo form transformation of *Z*1/F^−^ and *Z*2/F^−^ is reversible and switching cycles can be repeated several times in both directions, with only slight sign of photodegradation in *Z*1/F^−^ photochromic system (Fig. 2 or S7†). In contrast to *Z*1/F^−^, the *Z*2/F^−^ photoswitching cycles can be repeated multiple times (up to 40 cycles were tried) without any marked signs of fatigue and with clear isosbestic point at approximately 480 nm (Fig. S7†). Because only *Z*1 isomer undergoes *Z*-to-*E* photoisomerization, we assume that the sign of photodegradation in *Z*1/F^−^ photochromic system results from the *Z*1-to-*E*1 photoprocess.

Bidirectional photoswitching between the hydrazone and the azo form is possible without using UV light and the *Z*1/F^−^ and *Z*2/F^−^ photochromic systems therefore can be clearly classified as Vis–Vis photoswitches, contrary to most of the common classes of photochromic compounds (with UV-Vis photochromic character).

Transient absorption spectra of the yellow hydrazone and the blue azo form generated in a nanosecond flash photolysis experiments exhibit two transient signals related to ground state depletion of the one form and corresponding production of the second form; confirming thus their photoswitching behavior (Fig. S10 and S11†). The absence of an additional triplet–triplet absorption signal in these spectra excludes the mutual azo-to-hydrazone photoconversion through the triplet excited states and simultaneously indicates that the reversible photoswitching occurs from the lowest excited singlet states S_1_ of hydrazone and azo forms.

The azo-to-hydrazone photoswitching behaviour of the *Z*1/F^−^ and *Z*2/F^−^ systems can be also observed in apolar CDCl_3_ (CHCl_3_) or benzene, although the switching amplitude in absorbance reaches its reasonable value only at approximately 1000 equivalents of TBA^+^F^−^ (Fig. S12–S14†). Thermal stability of the blue azo form of *Z*1/F^−^ system in CDCl_3_ is practically the same as in DMSO-*d*_6_ (*k* ∼1.1 × 10^−2^ min^−1^; *t*_1/2_–60 min). Similar photochromic behaviour can be observed also for *Z*2/F^−^ in benzene (Fig. S14†). In contrast to benzene, competitive photodegradation pathways were observed for the *Z*2/F^−^ in CDCl_3_, to a lesser extent in CHCl_3_ (Fig. S15†). We assume that the light initiated radical reactions could be responsible for this fast photodegradation of the *Z*2/F^−^ photochromic system. However, the detailed photodegradation mechanism needs deeper investigation.

The photoswitching behaviour can be also observed in presence of other strongly basic anions, for example TBA^+^CH_3_COO^−^ (Fig. S16†). Interestingly, facile photochromic behavior through a large anion concentration region can be observed in the presence of less basic Cl^−^ anion, although the amplitude of photoswitching (absorbance changes during the photoswitching cycle) decreases compared to the *Z*/F^−^ photochromic system (Fig. S17 and S18†). This behavior clearly indicates the crucial role of an anion basicity in stabilization of the blue form.

It should be noted here that the azo–hydrazone (keto–enol) photochromic behaviour of *Z*1/F^−^ and *Z*2/F^−^ can be observed only in aprotic solvents like DMF and DMSO, but not in MeCN or MeCN/H_2_O mixture, because the protic solvents shifts the azo–hydrazone tautomeric equilibrium significantly to the hydrazone form side. Interestingly, addition of strongly basic F^−^ anion to solution of the *E* hydrazone isomers leads to the same blue azo form as its addition to solutions of *Z* hydrazone isomers (Fig. S19 and S20†). Subsequent photoswitching behaviour then unexpectedly results from the mutual phototransformation of the above mentioned blue azo form and the *Z* hydrazone isomers (Fig. S21–S23†).

### Photoswitching mechanism

**Scheme 3:**
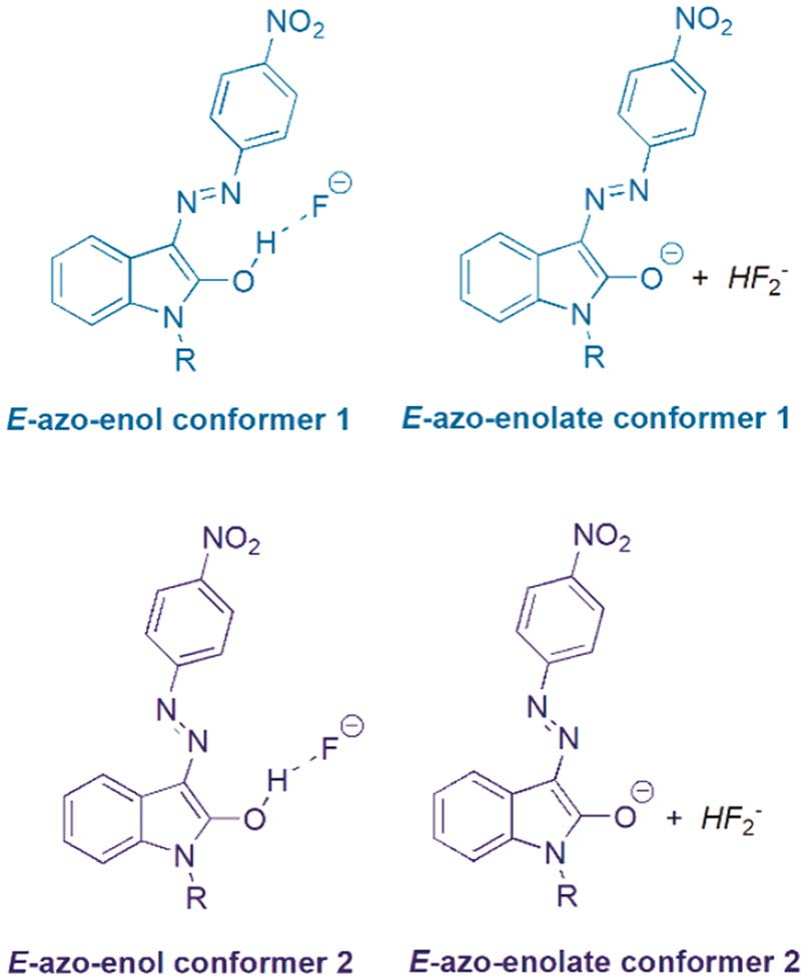
Two azo-enol (azo-enolate) conformers 1 and 2 derived from *Z* and *E* isomers of the studied isatin 4-nitrophenylhydrazones 1 and 2 after TBA^+^F^−^ addition.

The initial *Z* and *E* hydrazone isomers should afford different azo-enol (azo-enolate) conformers after basic anion addition. The *Z* isomer should lead to *E*-azo-enol (*E*-azo-enolate) conformer 1, whereas *E*-isomer should offer corresponding *E*-azo-enol (*E*-azo-enolate) conformer 2 (Scheme 3).

However, the final ^1^H NMR spectra of *E* and *Z* isomers in DMSO-*d*_6_ after TBA^+^F^−^ addition are identical (Fig. S19–S22†), although relatively slow conformational change of conformer 1 to conformer 2 is evident (Fig. S24–S27†). Only characteristic doublet signal at 8.35 ppm from isatin moiety related to *E*-azo-enol (*E*-azo-enolate) conformer 2 can be recorded in ^1^H NMR spectra of the both *Z*1 and *Z*2 solutions several minutes after TBA^+^F^−^ addition (Fig. 8 in ref. 39; signal of hydrogen “d’” in Fig. S19 and S20†). Gradual hypsochromic shift of the *Z*1 and *Z*2 absorption maxima after TBA^+^F^−^ addition also supports the equilibrium between the conformers Fig. S28.†^39^

**Scheme 4:**
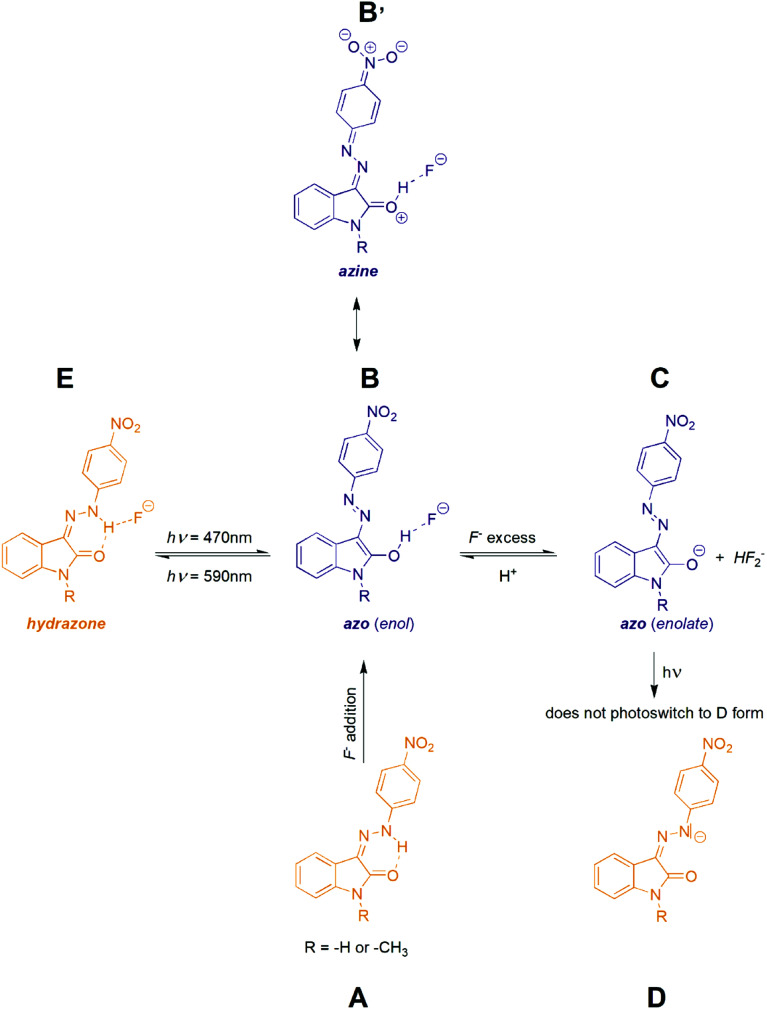
Photoswitching behaviour of the studied isatin 4-nitrophenylhydrazone *Z* isomers *Z*1 and *Z*2.

As indicated, the additon of F^−^ to initial *Z* hydrazone form (A) can stabilize blue azo form through hydrogen bonding (B) or can lead to deprotonation (azo anion C formation) with very similar UV-Vis spectrum (Scheme 4).^39^ Moreover, because of practically identical electron density (and thus shielding of protons) in B and C, also ^1^H NMR spectra of both forms B and C are almost identical. However, F^−^ anion excess (>2 equiv.) resulting to phenylhydrazone deprotonation is accompanied by formation of stable HF_2_^−^ anion with characteristic triplet at approximately 16.5 ppm with interaction constant of 120 Hz (Fig. S29†).

**Fig. 5:**
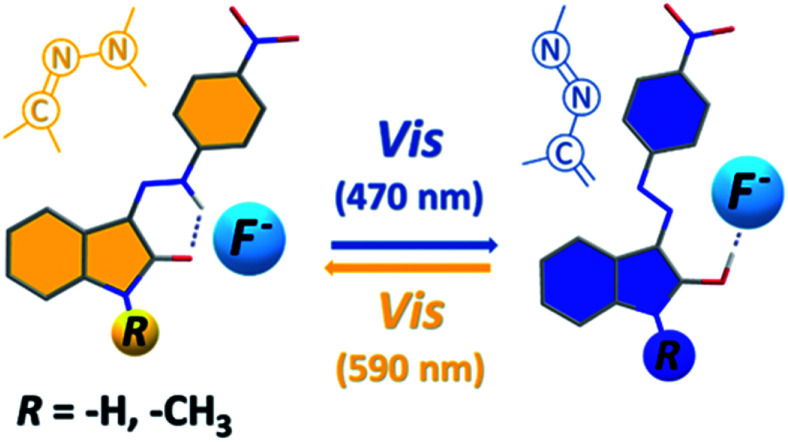
Structural changes related to photoswitching behaviour of the studied *Z*1/TBA^+^F^−^ and *Z*2/TBA^+^F^−^ photochromic systems (hydrazone-to-azo/azine photo-tautomerization process).

Interestingly, the 590 nm light irradiation of blue anionic form C in presence of significant F^−^ anion excess (∼10 equiv.) does not lead to previously observed photoswitching. Although this behaviour indicates photoswitching between hydrazone A and azo B forms, unchanged UV-Vis spectrum of C form at 590 nm irradiation could still result from fast back thermal reaction of anionic hydrazone form D. Such extremely fast back thermal reaction (isomerization) in a microsecond to a second timescale is commonly known in push–pull azobenzenes and was recently found also in push–pull azopyrimidines.^39^ However, absence of a fast signal decay in ultrafast UV-Vis kinetics measurement after 590 nm excitation (Fig. S30†) – together with an absence of ground state depletion signal at 590 nm in transient absorption spectrum of C (Fig. S10 and S11†) – exclude this fast back thermal reaction. Furthermore, addition of an acid (few drops of H_2_O, MeOH or CF_3_COOH) in the presence of the significant F^−^ anion excess regenerates the photoswitching behaviour of the whole system. Therefore, we assume that the photochromic behavior of *Z*1/F^−^ and *Z*2/F^−^ systems results from mutual hydrazone (keto) *E* and azo (enol) B form photoconversion rather than from photoswitching between the corresponding hydrazone (keto) D and azo (enolate) C anions (Scheme 4 and Fig. 5). However, the fast photoswitching of blue azo B form to yellow hydrazone (keto) *E* form and the presence of one isosbestic point indicate at least partial double-bond character of the C3(isatin)–N bond in the blue B form. Because structure of the blue B form can also be represented by the azine resonance form B′ (Scheme 4), the photoswitching behaviour of *Z*1/anion and *Z*2/anion photochromic systems can be classified as an unusual azo/azine-to-hydrazone photo-tautomerization process. As expected, excess of the acid leads to yellow neutral hydrazone form (without hydrogen bonding to F^−^), which does not photoswitch to corresponding blue neutral azo form.

### Polymer matrix

**Fig. 6:**
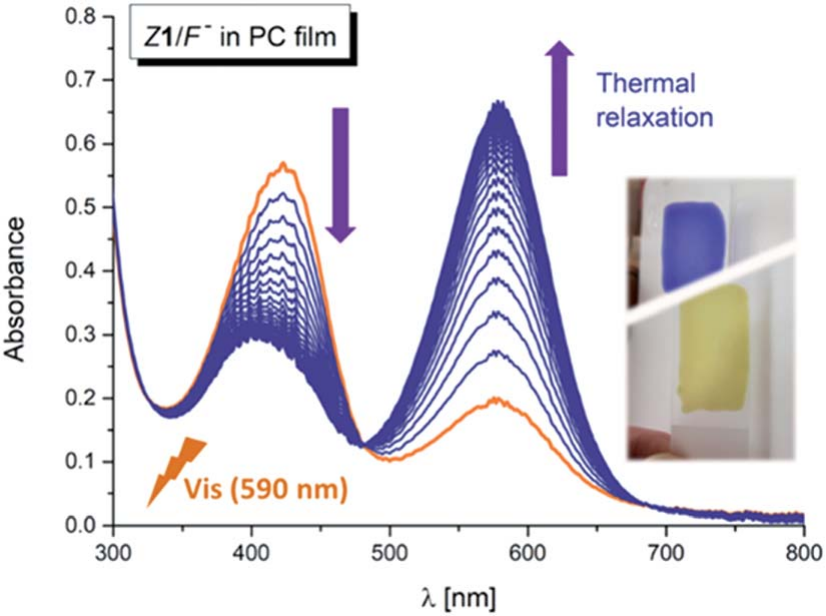
UV-Vis spectral changes of the *Z*1/TBA^+^F^−^ photochromic system in poly(propylene carbonate) thin polymer film – back thermal reaction after initial polymer film irradiation with light of 590 nm wavelength (*T* = 298.15 K).

**Fig. 7:**
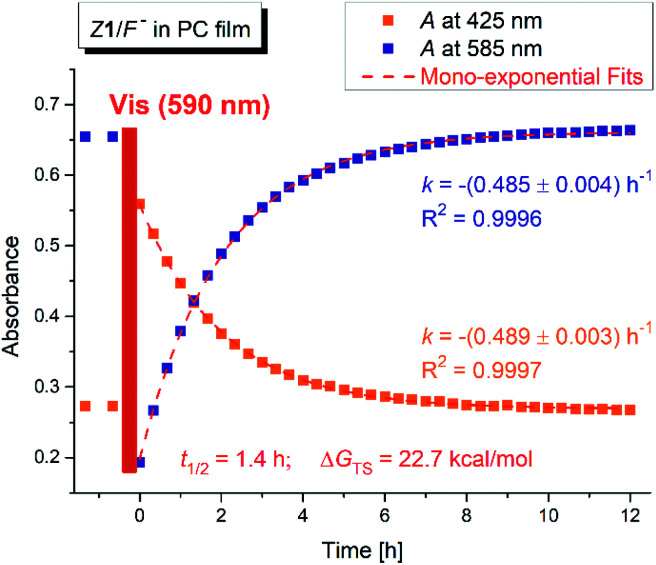
Absorbance changes of the *Z*1/TBA^+^F^−^ photochromic system in poly (propylene carbonate) thin polymer film after initial film irradiation with light of 590 nm wavelength (*T* = 298.15 K; *k* is the rate constant calculated from mono-exponential fit of concentration change of the corresponding form, *t*_1/2_ is the half-life of back thermal reaction and Δ*G*_TS_ is the Gibbs free energy of the transition state for this reaction).

Interestingly, the photochemical conversion of the yellow hydrazone form is significantly suppressed in poly(propylene carbonate) polymer matrix and prepared thin polymer film of the *Z*1/F^−^ system exhibits T-type photochromic properties (Fig. 6 and S31†). On the contrary to apolar solvents, the blue azo/azine form is thermodynamically more stable in the polycarbonate polymer matrix and the photochemically induced yellow polymer film (hydrazone form) returns thermally to its initial blue form with lifetime of 1.4 h at room temperature (Δ*G*_H–A_^‡^ = 95 kJ mol^−1^ = 22.7 kcal mol^−1^; Fig. 7 and S32†). As expected, the overall process is again reversible and switching cycles can be repeated.

## Conclusions

This paper examined the photochemical (photochromic) properties of two novel two-component photochromic systems based on the isatin 4-nitrophenylhydrazones and corresponding basic anions. The photoswitching behaviour of *Z*1/anion and *Z*2/anion photochromic systems can be classified as an unusual hydrazone-to-azo/azine photo-tautomerization process. Although the strongly basic F^−^ anion with the ability of stronger hydrogen bonding stabilizes the blue azo/azine form more efficiently, its excess results in complete blocking of photoswitching behavior. The appropriate basicity of the anion, together with the suitable counter anion partner selection, will therefore play a crucial role in optimization of the photoswitching behaviour of these systems in a concrete matrix (polymer, polyelectrolyte, *etc.*). The necessity of optimal hydrazone/anion ratio and optimal anion selection is therefore the main disadvantage of these photochromic systems. The role of the corresponding cation remains unknown yet.

## Conflicts of interest

There are no conflicts to declare.

## Supplementary Material

RA-009-C9RA02906K-s001
