# Supplementary material for: Tautomeric photoswitches: anion-assisted azo/azine-to-hydrazone photochromism
Source: RSC Adv. 2019 May 21;9(28):15910–6. doi: 10.1039/c9ra02906k (PMC9064339; doi:10.1039/c9ra02906k)
Supplement: RA-009-C9RA02906K-s001 [file RA-009-C9RA02906K-s001.pdf]

Electronic Supplementary Material (ESI) for RSC Advances.
This journal is © The Royal Society of Chemistry 2019

Tautomeric photoswitches: Anion-assisted *azo/azine*-to-*hydrazone* photochromism

J. Filo,[a] P. Tisovský,[a] K. Jakusová,[a] J. Donovalová,[a] M. Gáplovský,[b] A. Gáplovský[a], M. Cigáň,[a,*]

[a]*Institute of Chemistry, Faculty of Natural Sciences, Comenius University, Ilkovičova 6, SK-842 15 Bratislava, Slovakia*
[b]*Department of Pharmaceutical Chemistry, Faculty of Pharmacy, Comenius University, Odbojárov 10, SK-832 32 Bratislava, Slovakia*

* - marek.cigan@uniba.sk

Supporting Information

Table of contents:

EXPERIMENTAL SECTION

Synthesis

General procedure

Characterization of compounds

Anions

Preparation of thin polymer film

NMR spectroscopy

Optical methods

Determination of photochemical quantum yields

Nanosecond flash photolysis

Quantum-chemical calculations

Computational details (Cartesian coordinates of optimized geometries)

References

SUPPORTING SCHEMES, TABLES AND FIGURES

EXPERIMENTAL SECTION

Synthesis

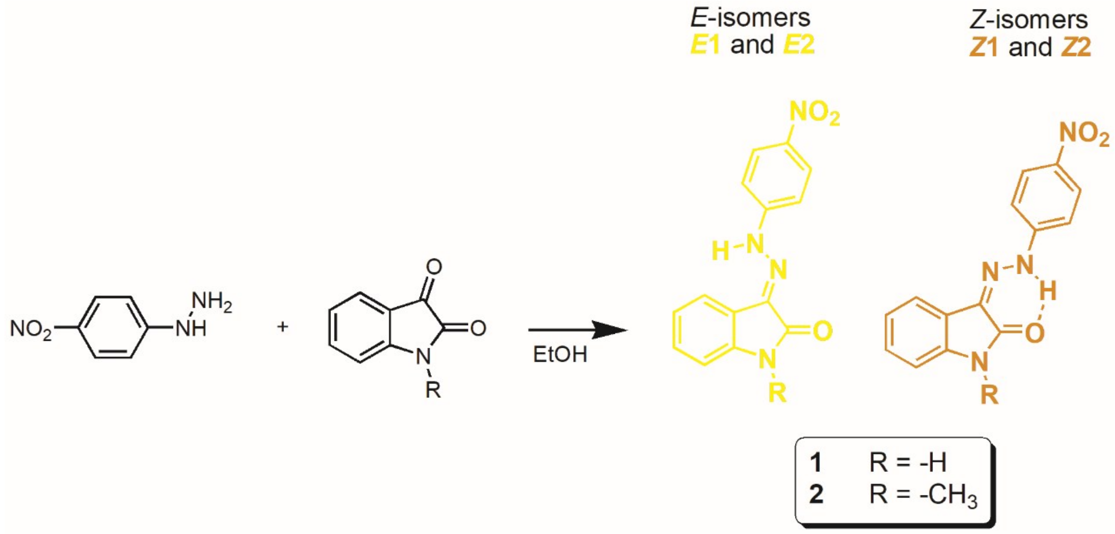

General Scheme: Synthesis of **1** and **2** in EtOH.

General procedure

A solution of isatin/1-methylisatin (3.4 mmol; both from Sigma Aldrich) and 4-nitrophenyl hydrazine (3.4 mmol, 520 mg; Sigma Aldrich) in ethanol (150 ml) was stirred at a specific temperature (see table) for specified time (see Table X1). After cooling, the precipitate was filtered, washed with cold ethanol (3 x 10 ml) and dried.

Table X1. Synthesis of *E* and *Z* isomers of isatin 4-nitrophenylhydrazones **1** and **2** in EtOH.

	Temp. [°C]	Reaction time [h]	Product
Isatin	78	24	***Z*1**
Isatin	25	48	***E*1**
1-methylisatin	78	24	***Z*2**
1-methylisatin	25	48	***E*2**

Characterization of compounds

(*Z*)-3-(2-(4-nitrophenyl)hydrazono)indolin-2-one (***Z*1**): orange solid, yield 76% M.P: 301-302°C.[1]

^{1}H NMR (600 MHz, DMSO-d_6) δ: 12.87 (s, 1H), 11.14 (s, 1H), 8.23 (d, *J* = 9.2 Hz, 2H), 7.65 – 7.57 (m, 2H), 7.31 (t, *J* = 7.7 Hz, 1H), 7.08 (t, *J* = 7.4 Hz, 1H), 6.93 (d, *J* = 7.8 Hz, 1H).

^{13}C NMR (75 MHz, DMSO) δ 162.66, 148.19, 141.42, 140.96, 131.66, 129.96, 122.09, 120.41, 119.58, 113.86, 110.69.

(*E*)-3-(2-(4-nitrophenyl)hydrazono)indolin-2-one (***E*1**): yellow solid, yield 73% M.P.: 281-282°C.

^{1}H NMR (600 MHz, DMSO-d_6) δ: 10.95 (s, 1H), 10.71 (s, 1H), 8.28 (d, J = 9.2 Hz, 1H), 8.23 (d, J = 7.6 Hz, 1H), 7.65 (d, J = 9.2 Hz, 2H), 7.37 (t, J = 7.7 Hz, 1H), 7.09 (t, J = 7.5 Hz, 1H), 6.92 (d, J = 7.7 Hz, 1H).

^{13}C NMR (150 MHz, DMSO-d_6) δ: 165.37, 150.35, 143.16, 141.88, 134.22, 131.92, 126.00, 125.49, 121.85, 116.20, 115.03, 110.81.

Elemental analysis (%) for $C_{14}H_{10}N_4O_3$: calculated C 59.57, H 3.57, N 19.85; found C 59.61, H 3.54, N 19.80.

(*Z*)-1-methyl-3-(2-(4-nitrophenyl)hydrazono)indolin-2-one (***Z*2**): orange solid, yield 57%, M.P.: 272-273 °C.[2]

1H NMR (300 MHz, DMSO-d_6) δ: 12.85 (s, 1H), 8.26 (d, J = 9.2 Hz, 1H), 7.67 (d, J = 9.1 Hz, 2H), 7.43 (td, J = 7.8, 1.2 Hz, 1H), 7.17 (dd, J = 7.2, 5.5 Hz, 1H), 3.28 (s, 2H).

^{13}C NMR (150 MHz, DMSO-d_6) δ: 160.81, 148.20, 142.30, 141.59, 132.06, 131.24, 123.99, 122.77, 119.72, 119.49, 119.12, 114.21, 113.83, 109.61, 25.52.

(*E*)-1-methyl-3-(2-(4-nitrophenyl)hydrazono)indolin-2-one (***E*2**): orange solid, yield 55%, M.P.: 230-231°C.

1H NMR (300 MHz, DMSO-d_6) δ: 11.02 (s, 1H), 8.28 (d, J = 7.2 Hz, 2H), 7.66 (d, J = 9.2 Hz, 1H), 7.46 (t, J = 7.6 Hz, 1H), 7.15 (dd, J = 19.9, 7.7 Hz, 2H), 3.21 (s, 2H).

^{13}C NMR (75 MHz, DMSO-d_6) δ: 163.58, 149.78, 143.79, 141.51, 132.95, 131.36, 125.52, 124.70, 121.87, 115.05, 114.64, 109.00, 25.92.

Elemental analysis (%) for $C_{15}H_{12}N_4O_3$: calculated C 60.81, H 4.08, N 18.91; found C 60.78, H 4.10, N 18.87.

Anions

All anions were added in the form of tetrabutylammonium (*TBA*$^+$) salts purchased from Sigma-Aldrich (USA), and used without further purification.

Preparation of thin polymer films

Poly (propylene carbonate) thin polymer films of two-component ***Z*1**/*F*$^-$ system were prepared by casting 1 mL chloroform solution of polymer (22 g/100 mL) containing the appropriate amount of isatin 4-nitrophenylhydrazone ***Z*1** and tetrabutylammonium fluoride onto a large Teflon plate or directly onto the 3×7 cm^2 quartz plate (***Z*1**:*TBA*$^+$*F*$^-$~1:1.3). The solvent was evaporated slowly. Remaining solvent was not additionally removed from the polymer. Poly (propylene carbonate) was purchased from Sigma-Aldrich, St. Louis, MO, USA, Mn ~ 50,000 by GPC). Final concentration of ***Z*1** in chloroform solution was ~ 5×10^{-4} mol dm^{-3}.

NMR spectroscopy

All NMR experiments were recorded on Varian VNMRS 300MHz or 600MHz spectrometer in 5mm NMR tube.

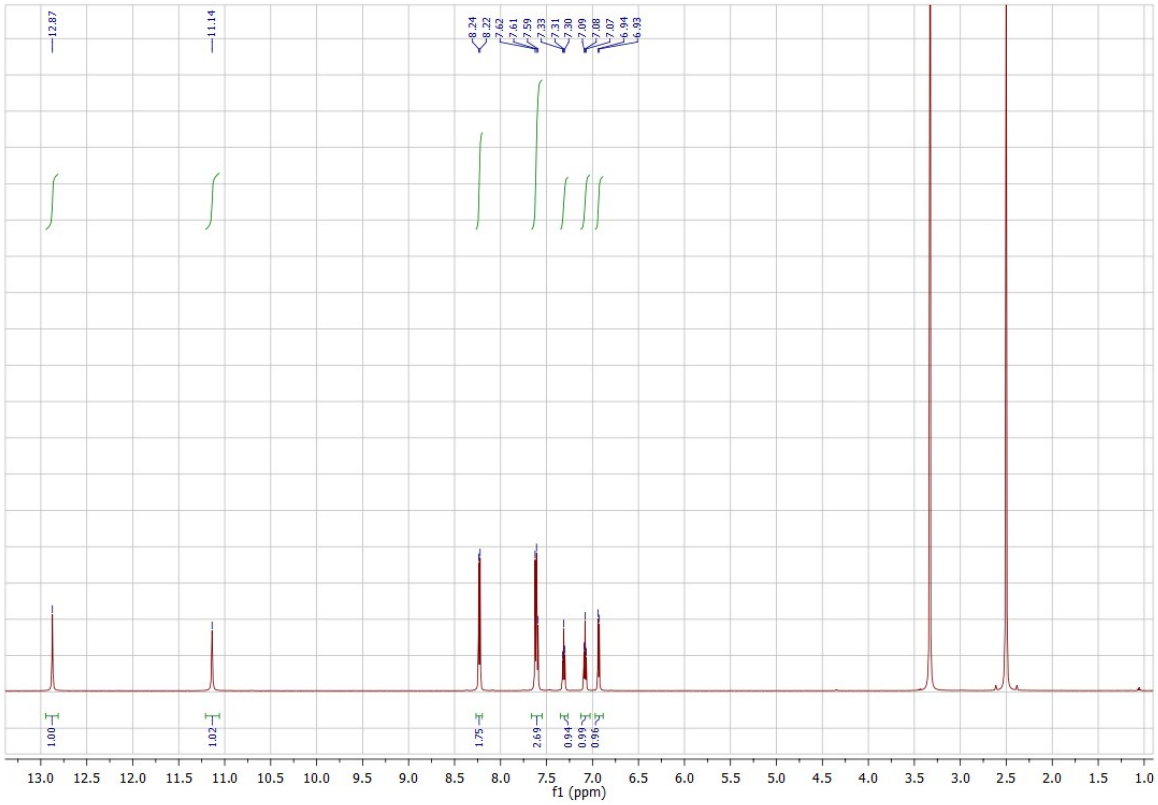

Fig. SXA. ^{1}H NMR spectrum of ***Z*1**.

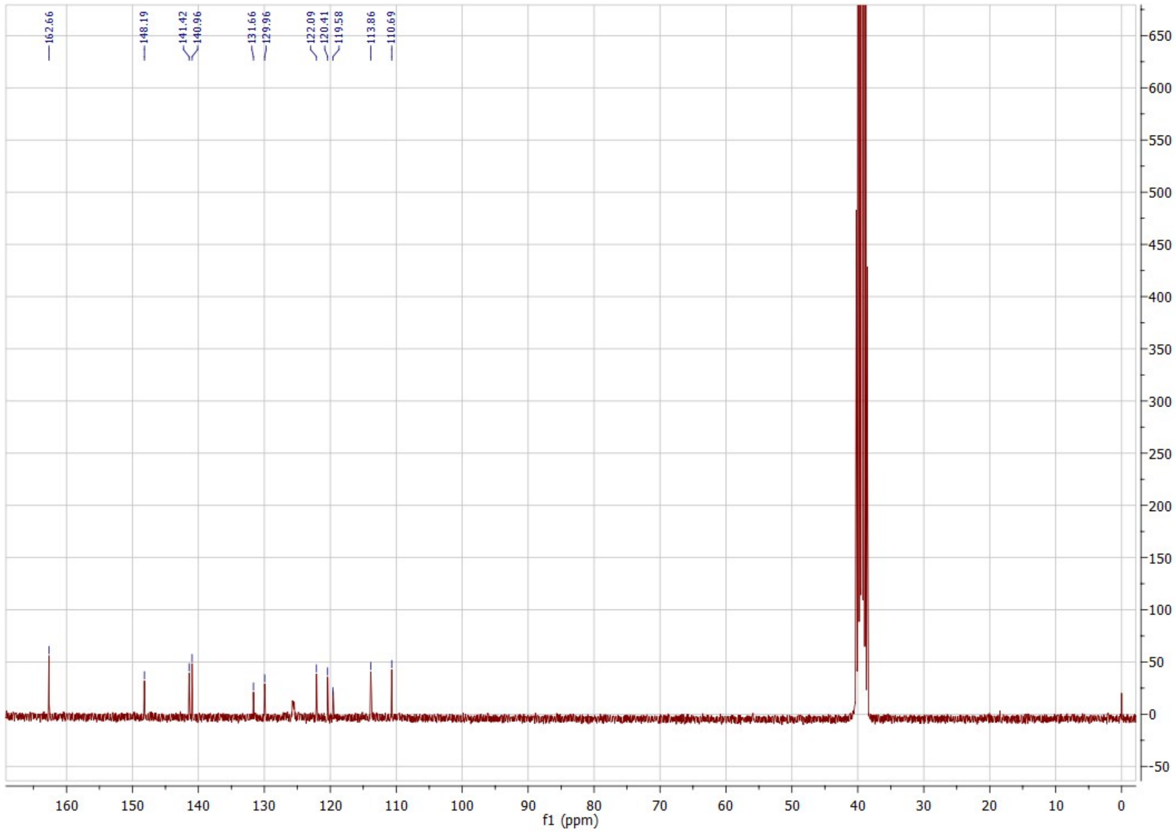

Fig. SXB. ^{13}C NMR spectrum of ***Z*1**.

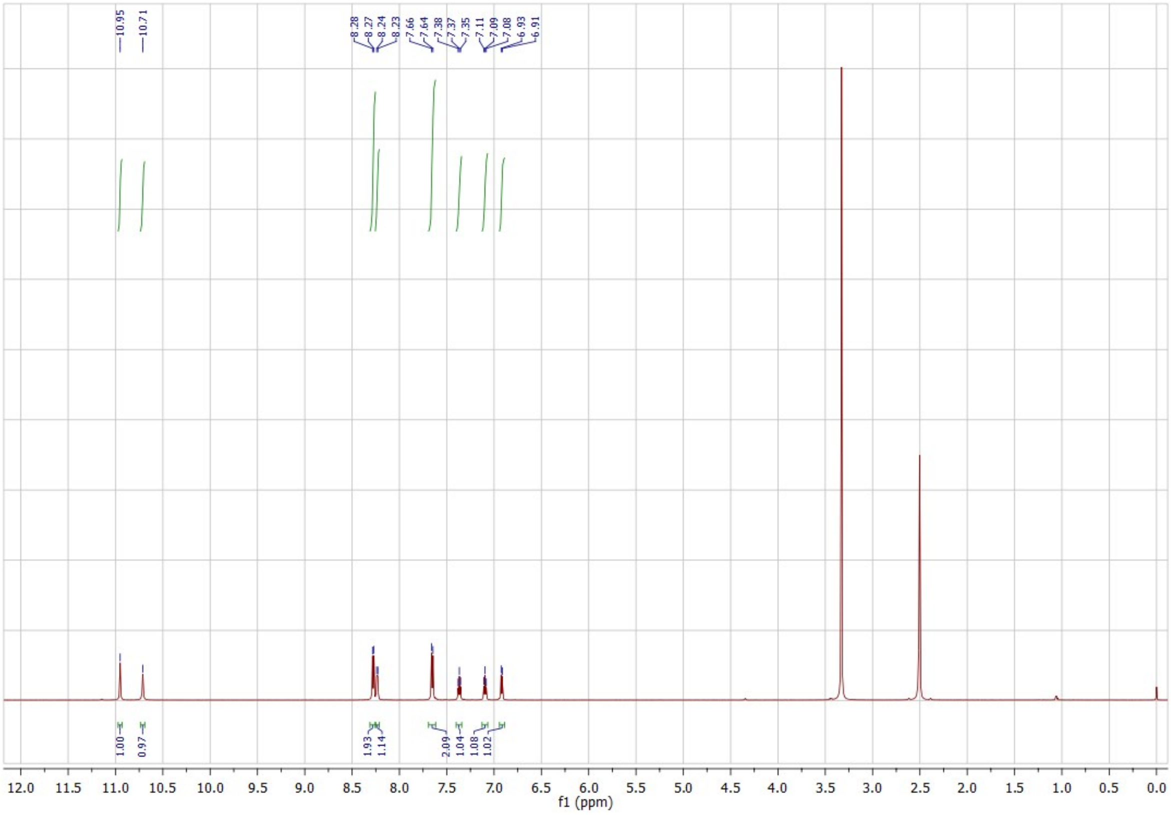

Fig. SXC. ^{1}H NMR spectrum of ***E*1**.

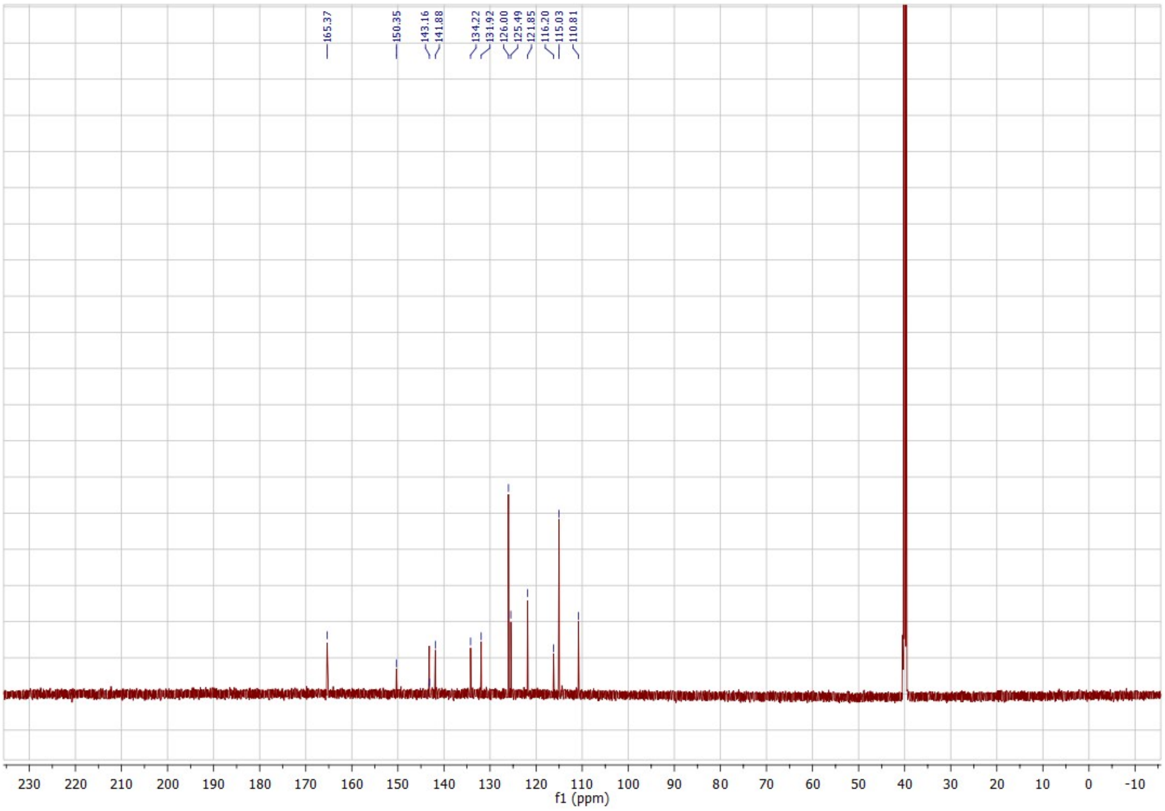

Fig. SXD. ^{13}C NMR spectrum of ***E*1**.

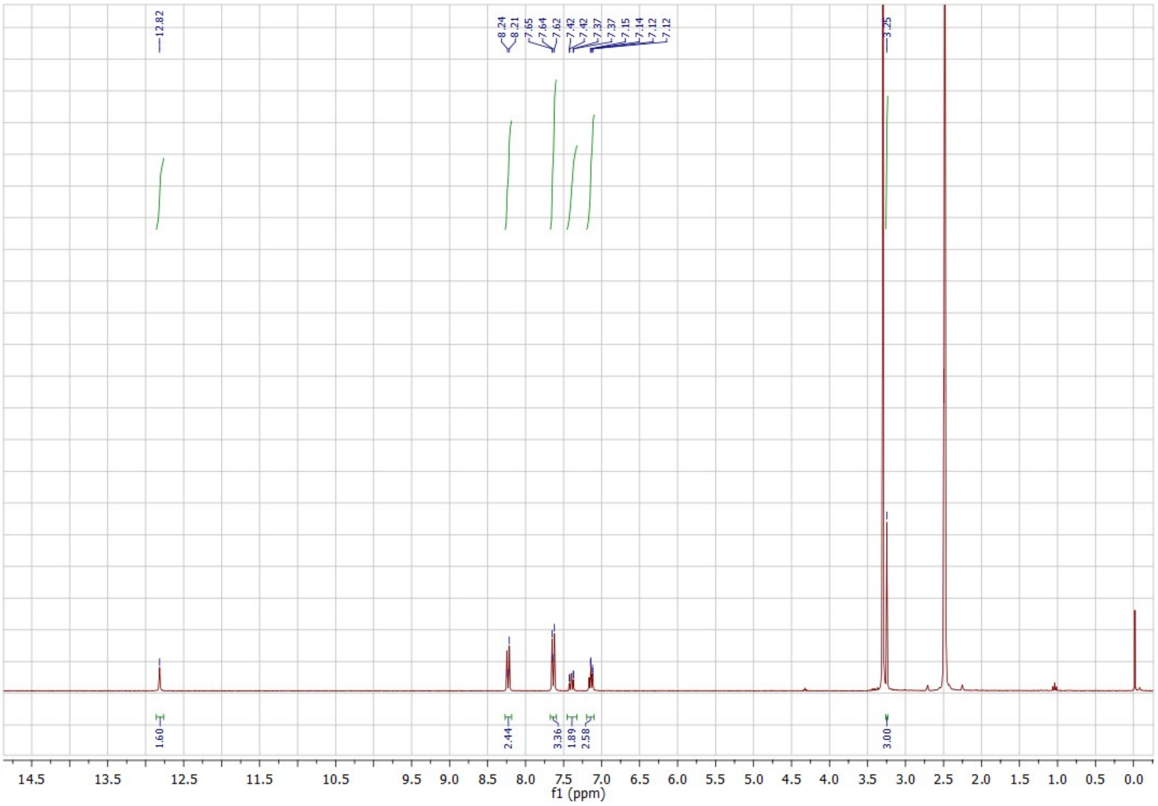

Fig. SXE. ^{1}H NMR spectrum of ***Z*2**.

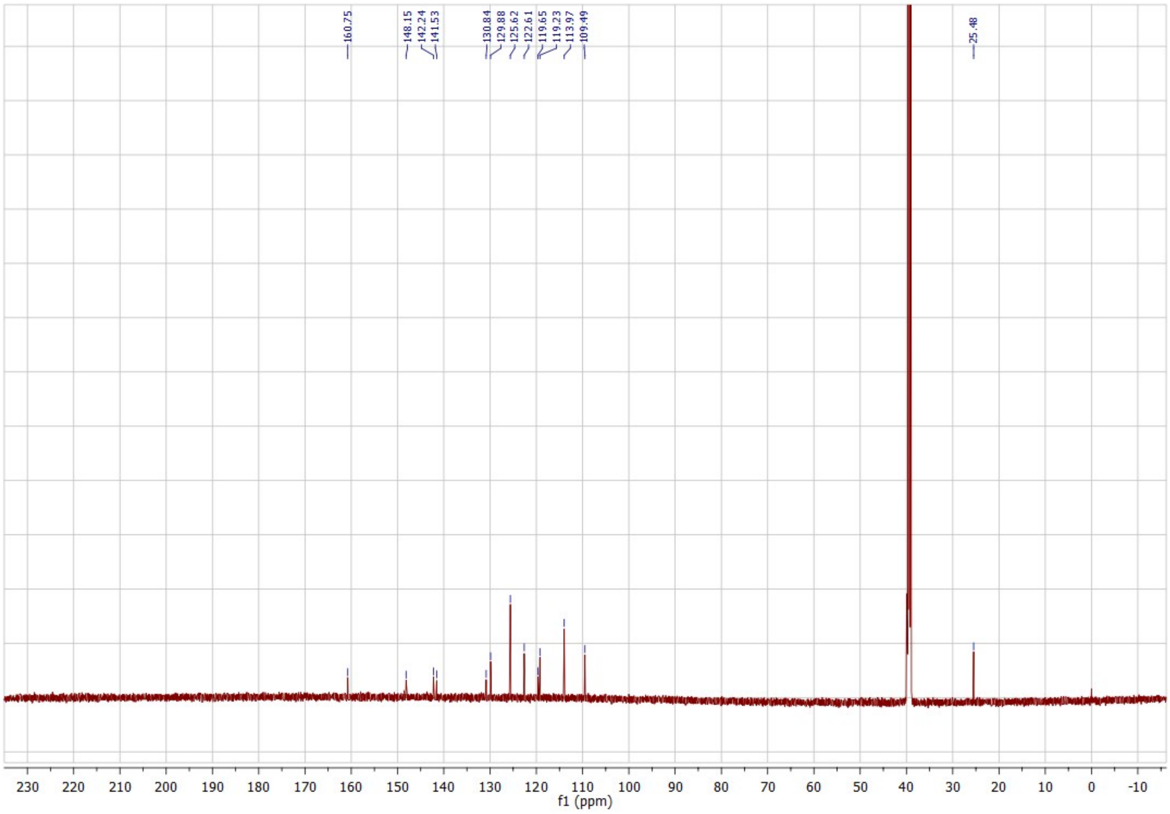

Fig. SXF. ^{13}C NMR spectrum of ***Z*2**.

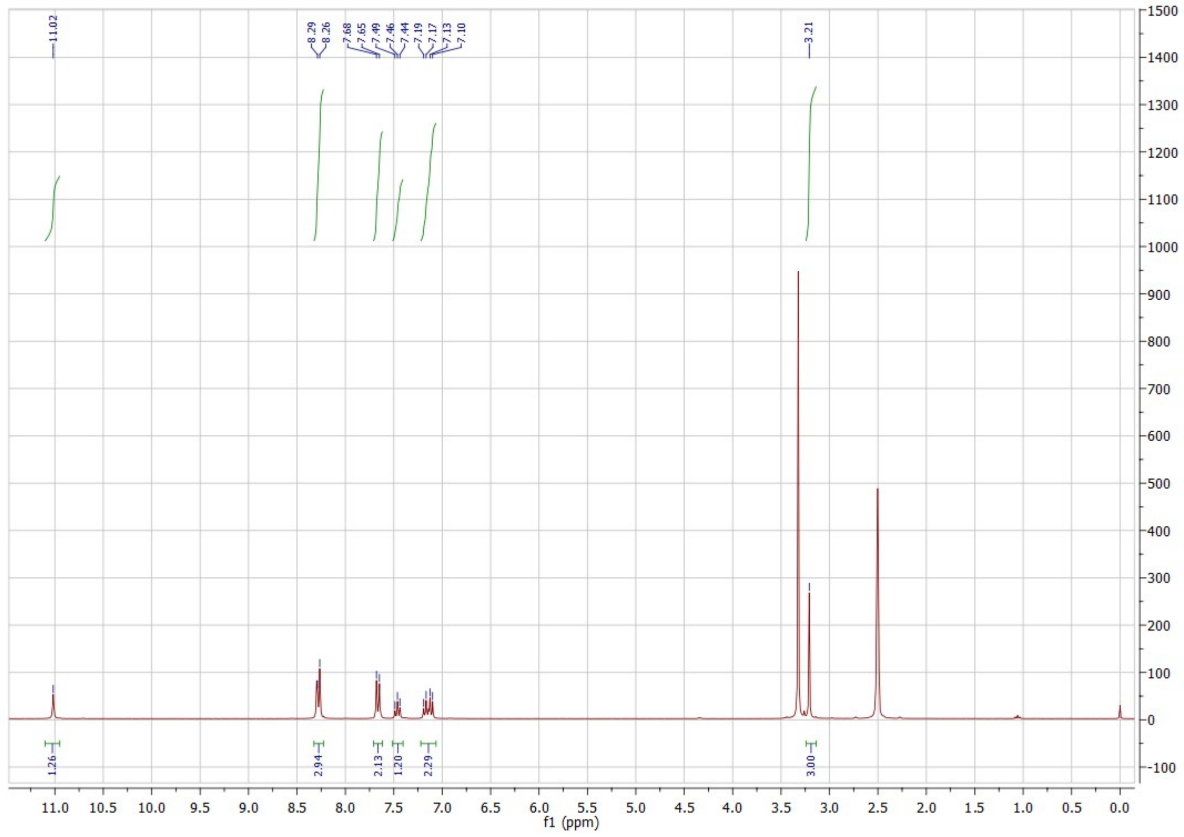

Fig. SXG. ^{1}H NMR spectrum of ***E*2**.

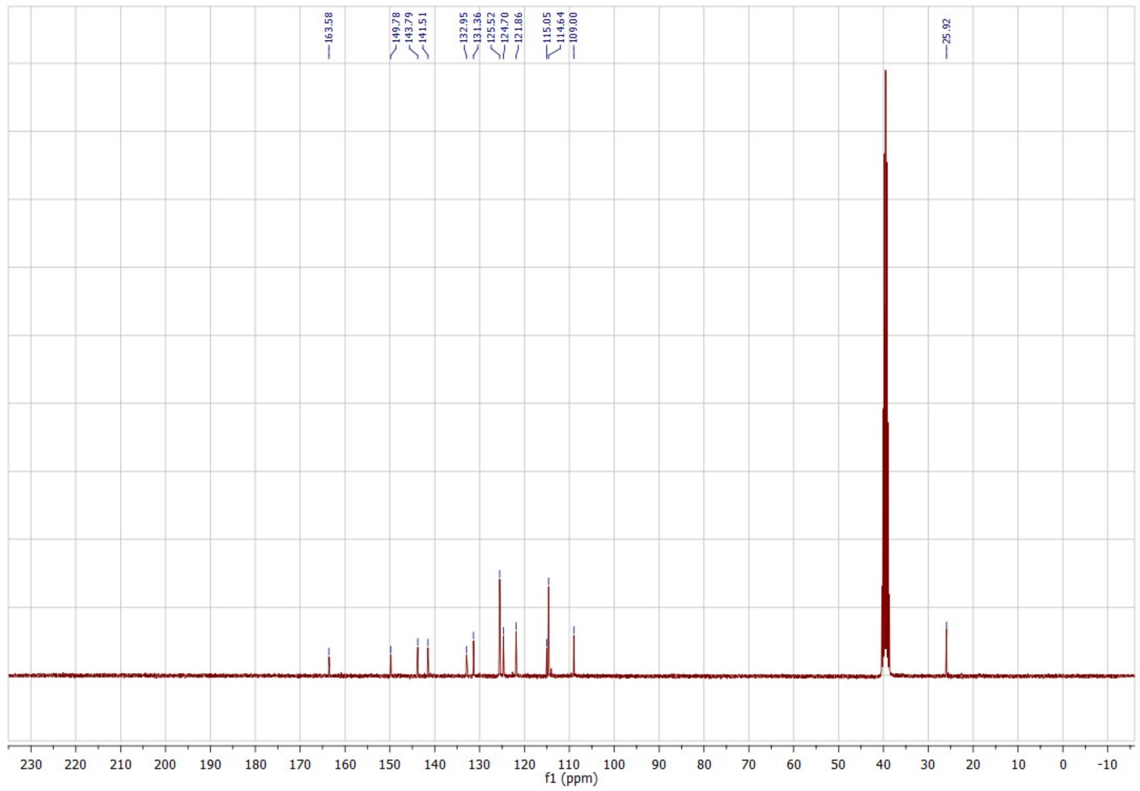

Fig. SXH. ^{13}C NMR spectrum of ***E*2**.

Optical methods

Electronic absorption spectra were obtained on a Agilent 8453 diode array spectrophotometer (Hewlett Packard, USA). The solvents used were NMR-spectroscopy (DMSO-d_6 and $CDCl_3$; Eurisotop, France) and UV-spectroscopy grade (DMF, 99.8% for spectroscopy, Acros Organics, UK; $CHCl_3$, Uvasol®, Merck, Germany) and were used without further purification. Solution fluorescence was measured in a 1 cm cuvette with FSP 920 (Edinburgh Instruments, UK) spectrofluorimeter in a right-angle arrangement. The fluorescent quantum yield (Φ_F) of the compounds studied in solution was determined by equations (1) and (2) using an integrating sphere (Edinburgh Instruments):

$$\Phi_F^X = \frac{L_{Sam}}{E_{Ref} - E_{Sam}}(\%) \tag{1}$$

corrected to re-absorption by:

$$\Phi_F = \frac{\Phi_F^X}{1 - a + a\Phi_F^X/100}\,(\%) \tag{2}$$

where L_{Sam} is the area under the detected spectrum in the part of the spectrum where sample emission occurs, E_{Ref} is area under the reflection part of the detected spectrum using the pure solvent as reference material (diffuse reflectance), E_{Sam} is area under the reflection part of the detected spectrum after absorption by the sample and a is the reabsorbed area. Transient absorption spectra were measured on a Flash photolysis LP980-Spectrometer (Edinburgh Instruments; λ_{exc} = 355 nm – Nd/YAG laser).

Irradiation experiments were performed at 25°C in the dark with 470 nm and 590 nm LED Array Light Sources (LIU470A and LIU590A; Thorlabs; Fig. SXI and SXJ), with intensity of 4.0 mW/cm^2 and 1.4 mW/cm^2 and and total output power of P = 253 mW and P = 109 mW, respectively.

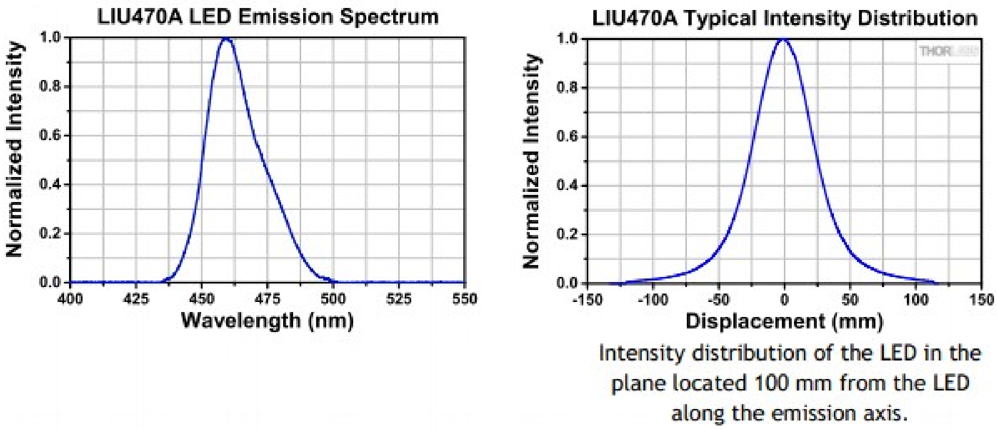

Fig. SXI. Emission spectra and typical intensity distribution of used LED Array Light Source (LIU470A; Thorlabs).

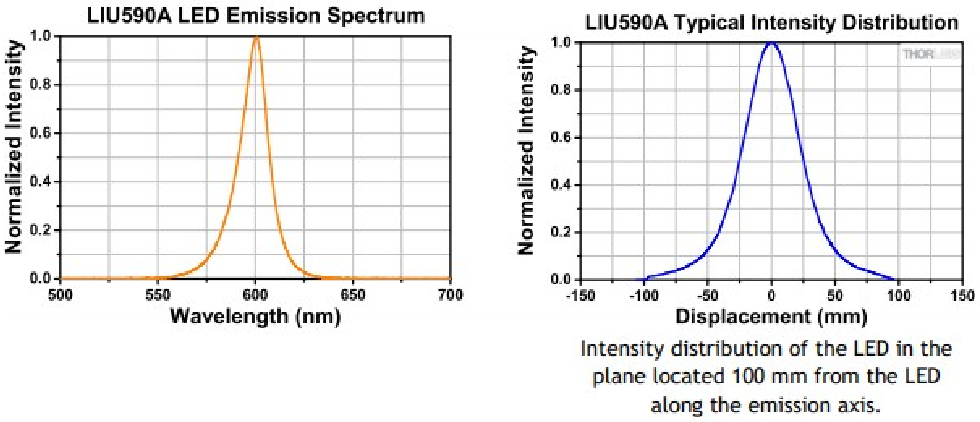

Fig. SXJ. Emission spectra and typical intensity distribution of used LED Array Light Source (LIU590A; Thorlabs).

All quantitative photochemical measurements were performed at 25°C in the dark, with either 370 405 nm or 591 nm epoxy-encased LED diodes (LED405E and LED591E; Thorlabs) as light sources with optical power of P=10 mW and P=2 mW, respectively. Photochemical measurements were performed using the apparatus described elsewhere (Fig.7 in reference [3]) without ultrasonic horn H and lens L1 and using a Thorlabs PM16-140 - USB Power Meter (Integrating Sphere Sensor, FC Fiber Adapter, Si, 350 - 1100 nm, 500 mW Max) fot initial and transmitted light intensity determination. The light sources were four 405 nm or four 591 nm LED diodes Thorlabs with overall incident photon flux $I_0=4.2\pm0.2\times10^{-5}$ mol s^{-1} dm^{-3} and $I_0=1.13\times10^{-5}$ mol s^{-1} dm^{-3}, respectively. The actual concentration of the blue *azo* and yellow *hydrazone* forms in freshly prepared air-saturated solutions during irradiation in a 1 cm quartz fluorescence cuvette were measured spectrophotometrically in right-angle arrangement (Agilent 8453; no interference with the irradiation LED light beam was observed). The photostationary state composition was determined using ^{1}H NMR spectroscopy.

Determination of photochemical quantum yields

The photochemical quantum yields of the mutual yellow *hydrazone* and blue *azo* form transformation ($\Phi_{H\text{-}A}$ and $\Phi_{A\text{-}H}$) of the studied isatin 4-nitrophenylhydrazones **1** and **2** were determined by equations (S1) and (S2) at low photochemical conversion (to eliminate the effect of back photoreaction)[4]:

$$\phi = \frac{\int_{c_0}^{c_t} dc}{\int_0^t I_a dt} = \frac{\Delta c}{\int_0^t I_a dt} \qquad \text{(S1)}$$

$$\Delta c = \frac{\Delta A_\lambda}{\varepsilon_{A\,\lambda}} \tag{S2}$$

where: Δc is the concentration change in blue *azo* form, I_a is light intensity absorbed by the yellow *hydrazone* or blue *azo* form at the irradiation wavelength λ_{irr} using a monochromatic light source, ΔA_λ is the absorbance change of the blue *azo* form at its absorption maximum, $\varepsilon_{A\lambda}$ is the molar extinction coefficient of the blue *azo* form at its absorption maximum and t is the irradiation time. Because the yellow *hydrazone* form practically does not absorb in the long-wavelength region of the blue *azo* form light absorption, the concentration change Δc of the yellow *hydrazone* was also determined by equation (S2). The extinction coefficient $\varepsilon_{A\lambda}$ of the blue *azo* form at its absorption maximum can be calculated based on the photostationary state composition determined by ^{1}H NMR spectroscopy and the corresponding absorption spectrum by Eq. (S3):

$$\varepsilon_{A\,\lambda} = \frac{A_\lambda}{c_A} \tag{S3}$$

where: c_A is the concentration of the blue *azo* form.

The absorbed light intensity I_a during the mutual yellow *hydrazone* and blue *azo* form transformation at the corresponding irradiation wavelengths λ was determined using a Thorlabs PM16-140 - USB Power Meter (Integrating Sphere Sensor, FC Fiber Adapter, Si, 350 - 1100 nm, 500 mW Max) by Eq. (S6):

$$I_{a\,\lambda} = I_{0\,\lambda} - I_{T\,\lambda} \tag{S6}$$

The incident light intensity $I_{0\ 405}$ at 405 nm was determined by ferrioxalate (FE) actinometry (Fig. SXK), according to Eq. (15)[5]:

$$I_{0\,405} = \frac{I_{\mathrm{a,\,FE\,405}}}{1 - 10^{-A_{\mathrm{FE\,405}}}} = \frac{\left(\frac{dA_{\mathrm{FE\,390}}}{dt}\right)\frac{1}{\Phi_{\mathrm{FE\,405}} \cdot \varepsilon_{\mathrm{FE\,390}} \cdot l}}{1 - 10^{-A_{\mathrm{FE\,405}}}}, \tag{15}$$

where: $I_{a,FE465}$ is ferrioxalate actinometer absorbed photon flux, A_{FE405} is ferrioxalate absorbance at 405 using the 405 nm Thorlabs LED light source, ε_{FE390} is ferrioxalate molar extinction coefficient at 390 nm, Φ_{FE405} is quantum yield of ferrioxalate photochemical conversion at 405 nm and l is the path length. Ferrioxalate absorbance at 405 nm (A_{FE405}) was measured by the Thorlabs PM16-140 - USB Power Meter.

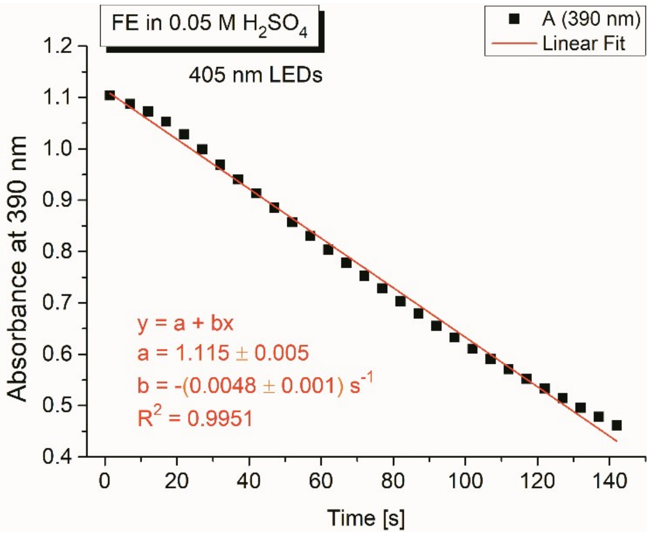

Fig. SXK. Decrease in absorbance of ferrioxalate (FE) actinometer solution at 390 nm in 0.05 mol dm^{-3} H_2SO_4 during irradiation with light of 405 nm wavelength (four LED405E diodes Thorlabs with individual optical power of P=10 mW).

Determined $I_{0\ 405}$=4.2±0.2×10^{-5} mol s^{-1} dm^{-3} is in good agreement with the theoretical value of $I_{0\ 405}$=3.9×10^{-5} mol s^{-1} dm^{-3} calculated from the manufacturer's incident optical power input of 40 mW (four epoxy-encased LED diodes Thorlabs LED405E) to cuvette volume of 3.5 mL.

Based on the absence of commercially available actinometer for the 590 nm wavelength, the incident light intensity $I_{0\lambda}$ at 590 nm was calculated from the manufacturer's incident optical power input of 8 mW (four epoxy-encased LED diodes Thorlabs LED591E) to cuvette volume of 3.5 mL ($I_{0\ 590}$ =1.13×10^{-5} mol s^{-1} dm^{-3}; 1W = 4.94×10^{-6} mol s^{-1} of photons at 590 nm).

Quantum-chemical calculations

The relative stabilities of isatin 4-nitrophenylhydrazone isomers were investigated using quantum-chemical calculations at the M062x 6-31+g(dp) level in vacuum. Stationary points were characterized as minima by computation of harmonic vibrational frequencies. All calculations were performed by Gaussian 16 program package.[6]

Computational details

Cartesian coordinates and electronic (*E*) and free (*G*) energies (atomic units).

Compound **1**

	***E*1 isomer**				***Z*1 isomer**		
C	4.05801	-2.49899	0.000133	C	-4.6411	-2.39784	0.000015
C	5.297736	-1.8624	0.000071	C	-5.73289	-1.52646	0.000011
C	5.393345	-0.46883	-3.6E-05	C	-5.55776	-0.13914	0.000003
C	4.216484	0.26274	-0.00008	C	-4.25765	0.338876	-2E-06
C	2.944973	-0.35778	-1.7E-05	C	-3.1486	-0.5259	0.000001
C	2.877927	-1.75091	0.000089	C	-3.33591	-1.90196	0.00001
C	1.964725	0.736286	-0.00009	C	-1.95979	0.319402	-4E-06
C	2.739413	2.031734	-0.00021	C	-2.4361	1.739051	-1.8E-05
N	4.071665	1.646906	-0.00019	N	-3.80616	1.66972	-1.2E-05
N	0.681301	0.809213	-7.1E-05	N	-0.73116	-0.08052	0.000001
N	-0.05002	-0.29475	0.000038	N	0.230903	0.829442	0.000006
C	-1.43936	-0.22184	0.000054	C	1.564872	0.446514	0.000003
C	-2.16985	-1.41867	0.000172	C	2.540896	1.455015	0.000009
C	-2.09885	1.015466	-4.5E-05	C	1.941898	-0.90456	-5E-06
C	-3.48466	1.045783	-2.6E-05	C	3.286891	-1.23824	-7E-06
C	-4.19447	-0.15134	0.000087	C	4.240736	-0.22491	-1E-06
C	-3.55471	-1.3862	0.000191	C	3.884663	1.120121	0.000007
O	2.325519	3.166642	-0.00029	O	-1.7505	2.754436	-2.7E-05
H	4.005171	-3.58189	0.000214	H	-4.81004	-3.46914	0.000022
H	6.205816	-2.45637	0.000107	H	-6.73995	-1.93057	0.000015
H	6.358645	0.026536	-8.5E-05	H	-6.40896	0.533775	0
H	1.9367	-2.29024	0.000137	H	-2.48018	-2.56928	0.000013
H	4.826314	2.31643	-0.00025	H	-4.39141	2.492126	-1.6E-05
H	0.385761	-1.2084	0.000123	H	-0.02576	1.818483	0.000014
H	-1.65172	-2.37342	0.000243	H	2.239921	2.498232	0.000016
H	-1.51752	1.928959	-0.00014	H	1.178437	-1.67165	-9E-06
H	-4.02356	1.985571	-0.0001	H	3.608441	-2.27274	-1.3E-05
H	-4.14275	-2.29579	0.000282	H	4.656596	1.879926	0.000012
N	-5.65862	-0.11268	0.000103	N	5.660047	-0.58333	-3E-06
O	-6.25322	-1.1782	0.000156	O	6.474212	0.325316	0.000012
O	-6.19698	0.981241	-5.1E-05	O	5.945849	-1.76929	-2E-06
E	-983.732996			E	-983.746256		
G	-983.550713			G	-983.560906		

Compound **2**

***E*2 isomer**

C	-4.66731	-2.34335	-1.9E-05
C	-5.74836	-1.45887	-1.6E-05
C	-5.55694	-0.0737	-4E-06
C	-4.25135	0.389293	4.78E-06
C	-3.15326	-0.48905	1.52E-06
C	-3.35663	-1.86273	-1E-05
C	-1.95384	0.340885	1.03E-05
C	-2.4117	1.76624	3.04E-05
N	-3.78264	1.714619	1.87E-05
N	-0.73083	-0.07559	0.000006
N	-0.46624	-1.3733	-1.3E-05
C	0.84619	-1.82498	-4.1E-05
C	1.068357	-3.21038	-5.5E-05
C	1.929101	-0.93364	-5.8E-05
C	3.222675	-1.43058	-8.9E-05
C	3.423832	-2.80749	-0.0001
C	2.361619	-3.70624	-8.5E-05
O	-1.7128	2.772422	5.23E-05
H	-4.84921	-3.41259	-2.8E-05
H	-6.76037	-1.85068	-2.3E-05
H	-6.40066	0.608591	-1.8E-06
H	-2.50816	-2.53934	-1.3E-05
H	-1.24444	-2.03542	2.48E-06
H	0.223545	-3.89277	-4.2E-05
H	1.743029	0.132744	-4.6E-05
H	4.079261	-0.76718	-0.0001
H	2.559556	-4.77147	-9.6E-05
N	4.792076	-3.32671	-0.00014
O	4.938768	-4.53786	3.06E-05
O	5.705943	-2.51857	2.49E-05
C	-4.6192	2.923361	2.77E-05
H	-5.42772	2.797553	-0.68945
H	-5.01039	3.086372	0.982525
H	-4.02843	3.765992	-0.29299
E	-1023.027713		
G	-1022.818267		

***Z*2 isomer**

C	4.278201	2.764916	0.000068
C	5.404785	1.940709	0.000114
C	5.287829	0.545854	0.00008
C	4.007919	0.01564	0.000055
C	2.863266	0.833958	0.000052
C	2.992998	2.215216	0.000009
C	1.716295	-0.06645	-0.00014
C	2.262912	-1.45998	-0.00024
N	3.628244	-1.33705	-0.00015
N	0.46988	0.272604	-0.00009
N	-0.44473	-0.6865	-0.00012
C	-1.7962	-0.37489	-6.3E-05
C	-2.71692	-1.43459	0.000079
C	-2.24605	0.953968	-0.00017
C	-3.6071	1.214233	-0.00013
C	-4.50506	0.150911	0.000013
C	-4.07662	-1.17296	0.000118
O	1.624939	-2.50941	-0.00033
H	4.402232	3.842394	0.00008
H	6.393912	2.387027	0.000161
H	6.166727	-0.09042	0.000033
H	2.110983	2.847451	-5.4E-05
H	-0.13533	-1.66051	0.000047
H	-2.3597	-2.45993	0.000163
H	-1.525	1.761111	-0.00027
H	-3.98436	2.229779	-0.00021
H	-4.80643	-1.97335	0.000231
N	-5.94128	0.432107	0.000057
O	-6.70544	-0.51912	0.000238
O	-6.29102	1.600928	0.000011
C	4.540192	-2.45804	0.000341
H	5.172495	-2.43574	0.892936
H	3.940985	-3.36907	0.001052
H	5.172063	-2.43697	-0.8926
E	-1023.041005		
G	-1022.829252		

References

1 J. Zheng, Y. Li, Y. Cui, J. Jia, Q. Ye, L. Han and J. Gao, *Tetrahedron*, 2015, **71**, 3802–3809.

2 J. C. Porter, R. Robinson and M. Wyler, *J. Chem. Soc.*, 1941, **0**, 620.

3 A. Gáplovský, Š. Toma and J. Donovalová, *J. Photochem. Photobiol. A Chem.*, 2007, **191**, 162–166.

4 P. Klán and J. Wirz, *Photochemistry of organic compounds : from concepts to practice*, Wiley, 2009, p 114.

5 T. Lehóczki, É. Józsa and K. Ősz, *J. Photochem. Photobiol. A Chem.*, 2013, **251**, 63–68.

6 Gaussian 16, Revision A.03, M. J. Frisch, G. W. Trucks, H. B. Schlegel, G. E. Scuseria, M. A. Robb, J. R. Cheeseman, G. Scalmani, V. Barone, G. A. Petersson, H. Nakatsuji, X. Li, M. Caricato, A. V. Marenich, J. Bloino, B. G. Janesko, R. Gomperts, B. Mennucci, H. P. Hratchian, J. V. Ortiz, A. F. Izmaylov, J. L. Sonnenberg, D. Williams-Young, F. Ding, F. Lipparini, F. Egidi, J. Goings, B. Peng, A. Petrone, T. Henderson, D. Ranasinghe, V. G. Zakrzewski, J. Gao, N. Rega, G. Zheng, W. Liang, M. Hada, M. Ehara, K. Toyota, R. Fukuda, J. Hasegawa, M. Ishida, T. Nakajima, Y. Honda, O. Kitao, H. Nakai, T. Vreven, K. Throssell, J. A. Montgomery, Jr., J. E. Peralta, F. Ogliaro, M. J. Bearpark, J. J. Heyd, E. N. Brothers, K. N. Kudin, V. N. Staroverov, T. A. Keith, R. Kobayashi, J. Normand, K. Raghavachari, A. P. Rendell, J. C. Burant, S. S. Iyengar, J. Tomasi, M. Cossi, J. M. Millam, M. Klene, C. Adamo, R. Cammi, J. W. Ochterski, R. L. Martin, K. Morokuma, O. Farkas, J. B. Foresman, and D. J. Fox, Gaussian, Inc., Wallingford CT, 2016.

SUPPORTING SCHEMES AND FIGURES

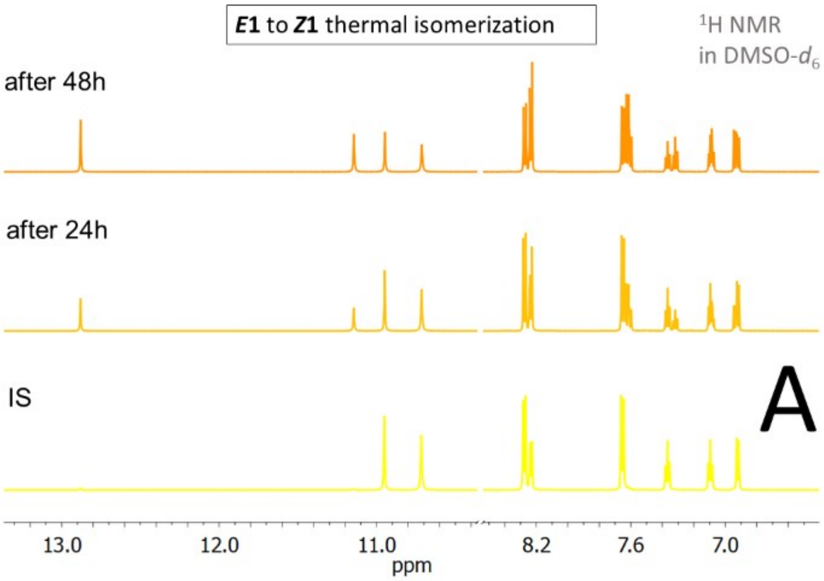

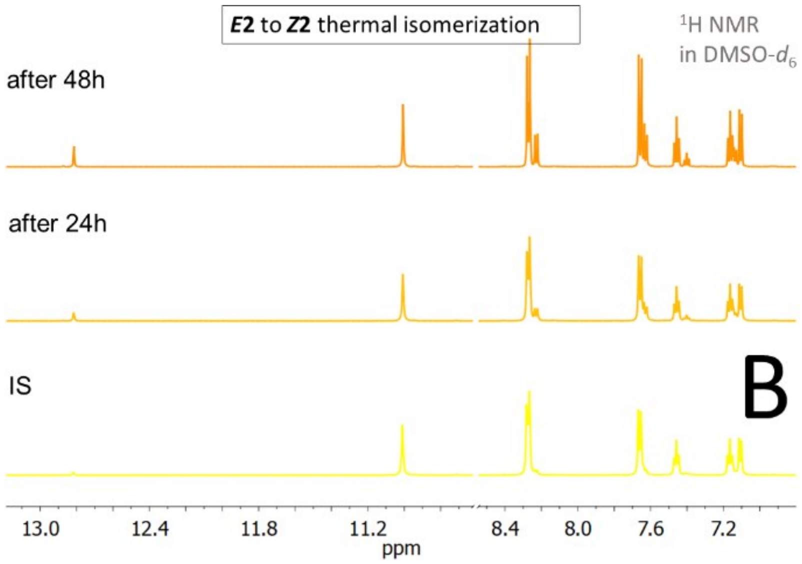

Fig. S1. ^{1}H NMR spectra of the ***Z*1** and ***Z*2** isomers during their slow thermal isomerization in DMSO-d_6 (T = 298.15 K; IS – initial state). New signals corresponding to the *E* isomers appeared.

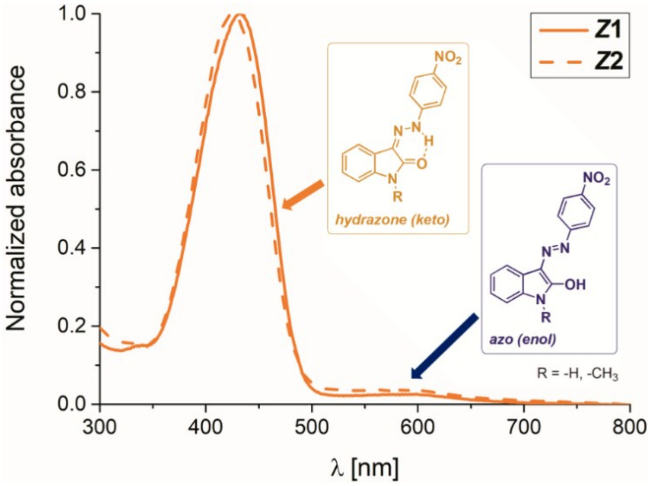

Fig. S2. UV-Vis spectra of the isatin 4-nitrophenylhydrazone *Z* isomers ***Z*1** and ***Z*2** in DMSO-d_6.

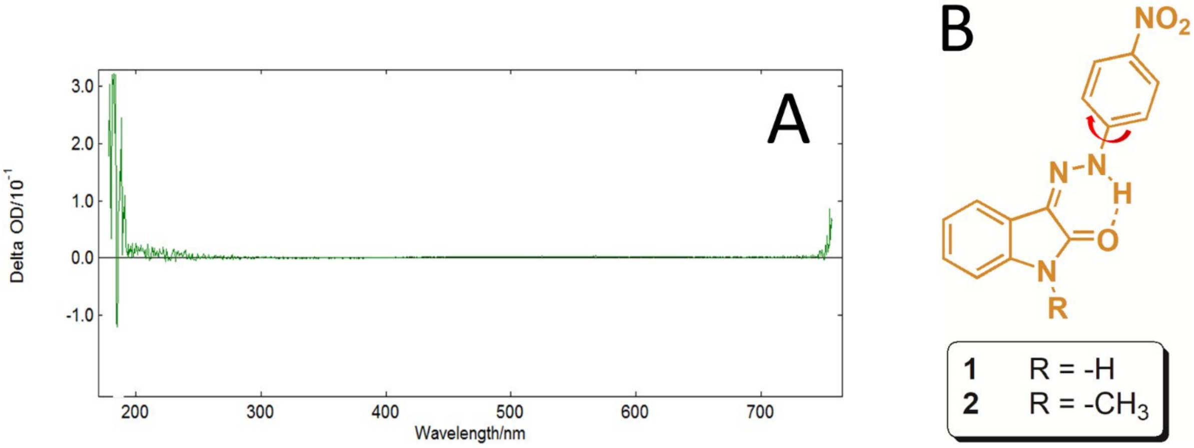

Fig. S3. (A) Transient absorption spectrum of the ***Z*1** in DMSO (Edinburgh Instruments Flash photolysis LP980-Spectrometer – nanosecond flash photolysis system; λ_{EX} = 355 nm; T = 298.15 K). Spectrum does not confirm the presence of any transient decay above 5ns in the region of 200-700 nm. (B) Intramolecular rotation of nitrophenyl ring in the ***Z*1** and ***Z*2** isomers.

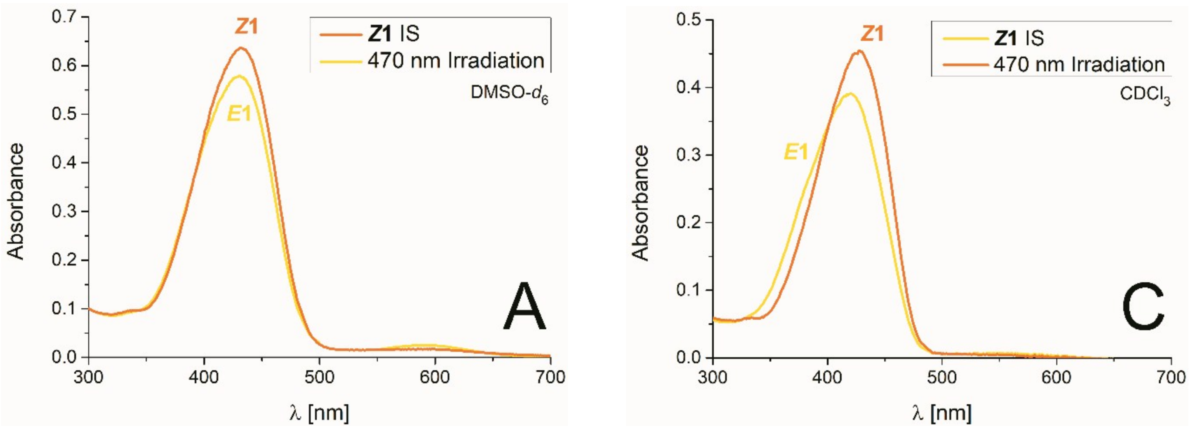

Fig. S4. UV-Vis spectral changes during the isatin 4-nitrophenylhydrazone ***Z1*** solution irradiation at 470 nm in DMSO-d_6 and $CDCl_3$ (initial *Z*-isomer concentration: c_{Z1} = 10^{-4} M; 0.2 cm cuvette; *T* = 298.15 K; IS – initial state).

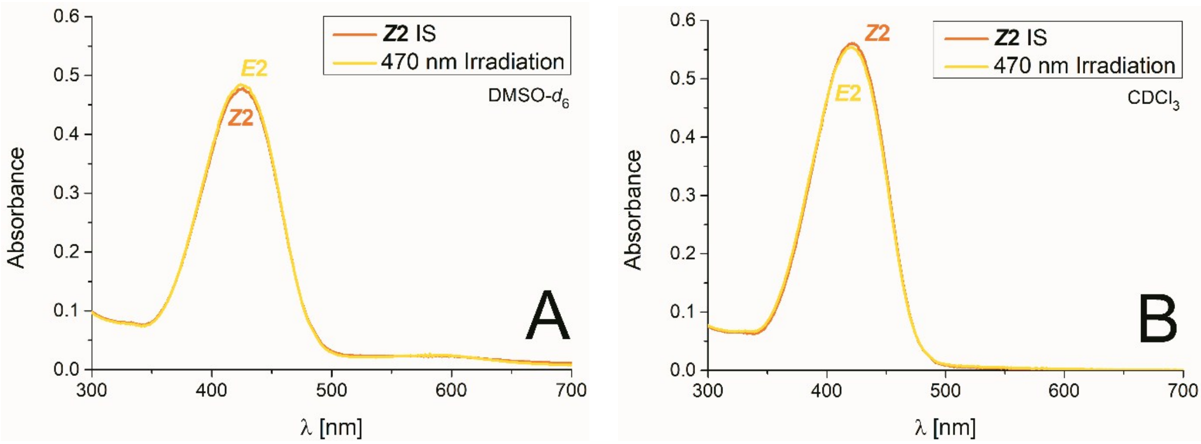

Fig. S5. UV-Vis spectral changes during the isatin 4-nitrophenylhydrazone ***Z2*** solution irradiation at 470 nm in DMSO-d_6 and $CDCl_3$ (initial *Z*-isomer concentration: c_{Z1} = 10^{-4} M; 0.2 cm cuvette; *T* = 298.15 K; IS – initial state).

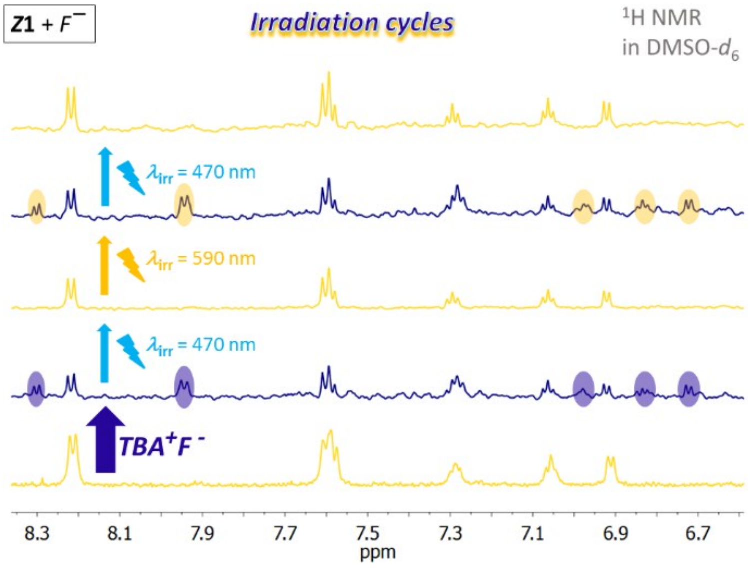

Fig. S6. ¹H NMR spectral change during altered irradiation of the ***Z1***/*TBA*$^+$*F*$^-$ photochromic system with light of 470 nm and 590 nm wavelength in DMSO-d_6 (T = 298.15 K).

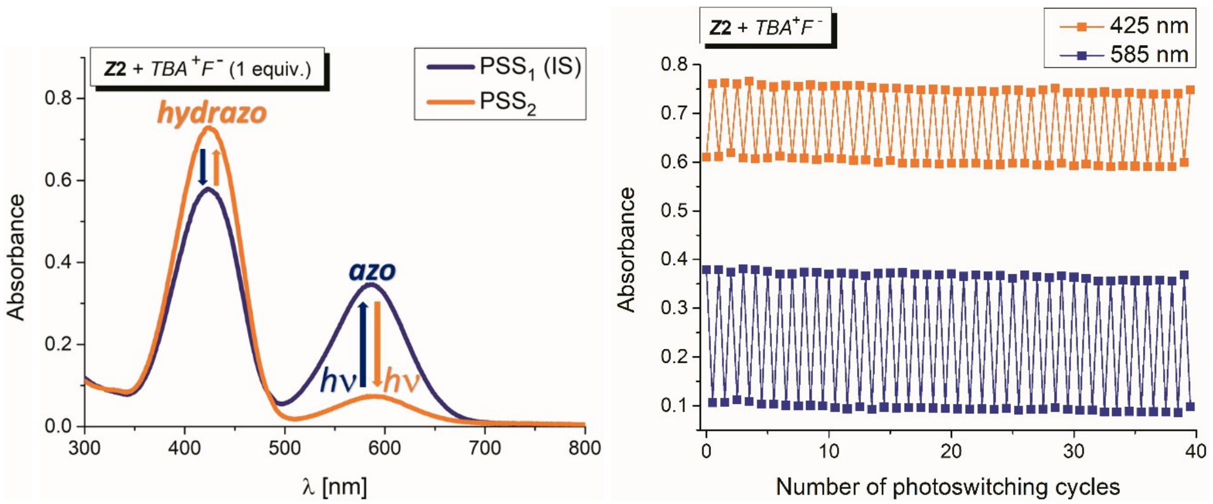

Fig. S7. UV-Vis spectral changes during altered irradiation of the ***Z2***/*TBA*$^+$*F*$^-$ photochromic system with light of 470 nm and 590 nm wavelength in DMSO-d_6 (initial *Z*-isomer concentration: c_{Z2} = 10^{-4} M; 0.2 cm cuvette; T = 298.15 K; IS – initial state; PSS – photostationary state).

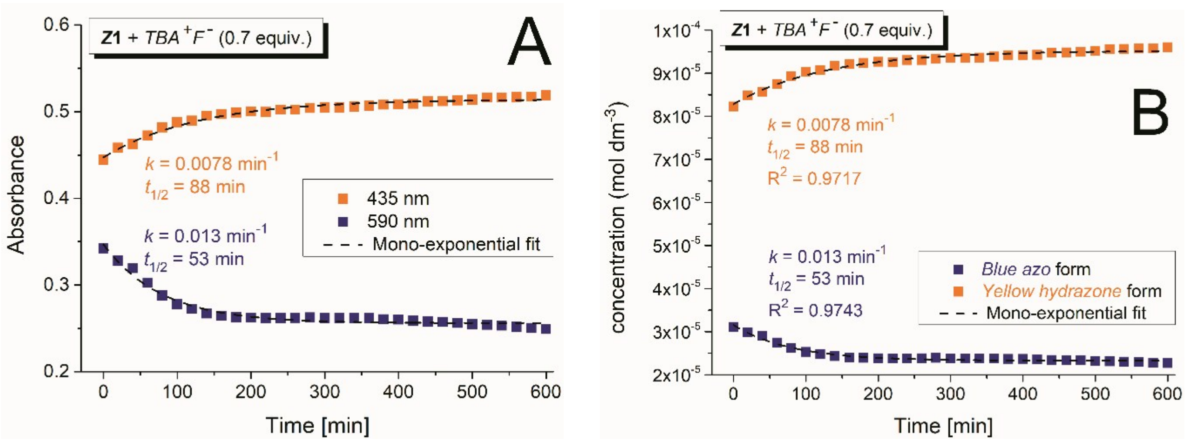

Fig. S8. Time-dependent absorbance (A) and concentration (B) changes of the ***Z1***/*TBA*$^+$*F*$^-$ photochromic system in DMSO-d_6 after initial irradiation with light of 470 nm wavelength in DMSO-d_6 (0.2 cm cuvette; T = 298.15 K; k is the rate constant calculated from mono-exponential fit of absorbance/concentration change of the corresponding form and $t_{1/2}$ is the corresponding thermal reaction half-life).

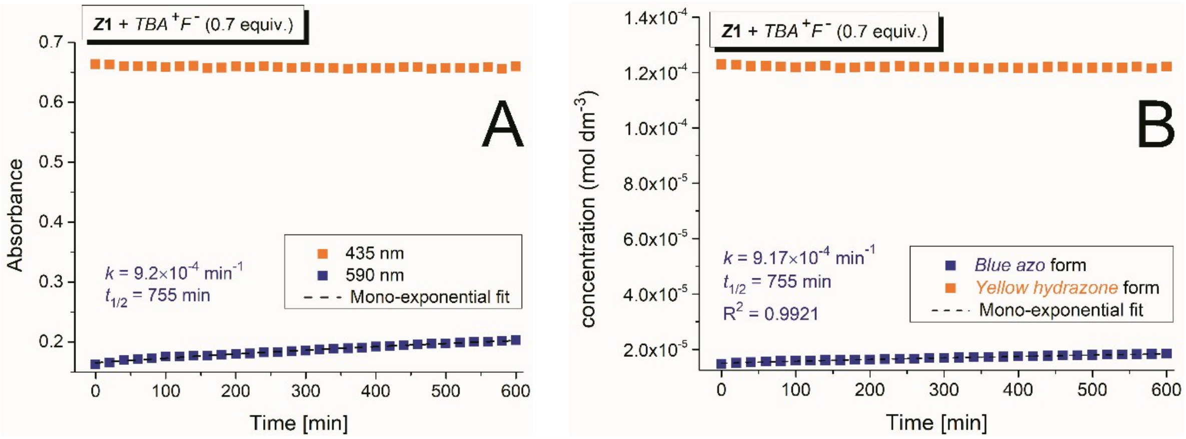

Fig. S9. Time-dependent absorbance (A) and concentration (B) changes of the ***Z1***/*TBA*$^+$*F*$^-$ photochromic system in DMSO-d_6 after initial irradiation with light of 590 nm wavelength in DMSO-d_6 (0.2 cm cuvette; T = 298.15 K; k is the rate constant calculated from mono-exponential fit of absorbance/concentration change of the corresponding form and $t_{1/2}$ is the corresponding thermal reaction half-life).

Nanosecond flash photolysis

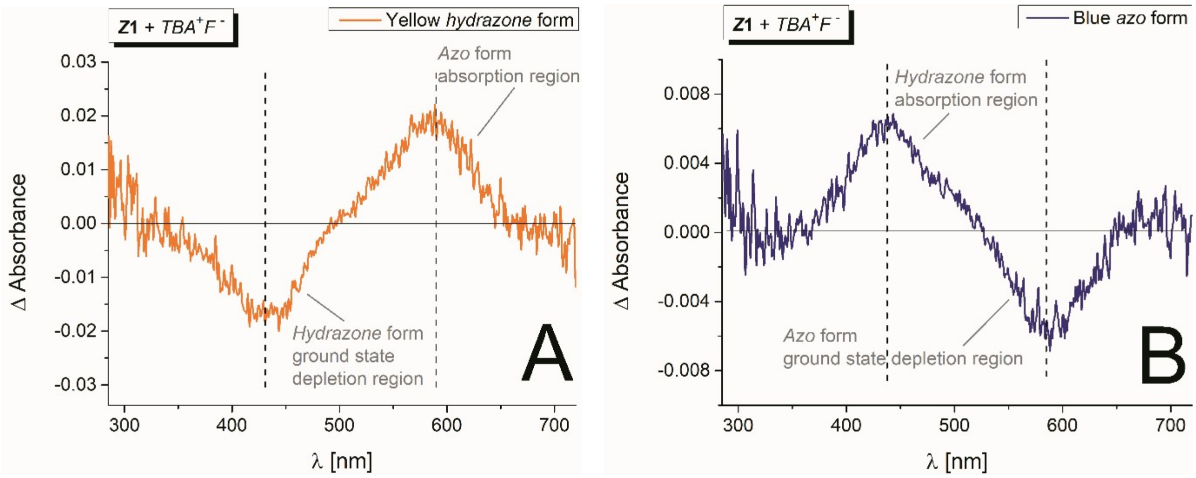

Fig. S10. Transient absorption spectra of the ***Z1***/*TBA*$^+$*F*$^-$ photochromic system in DMSO-d_6 after initial irradiation with light of (A) 590 nm and (B) 470 nm wavelength (λ_{exc} = 355 nm; 1 cm cuvette; T = 298.15 K).

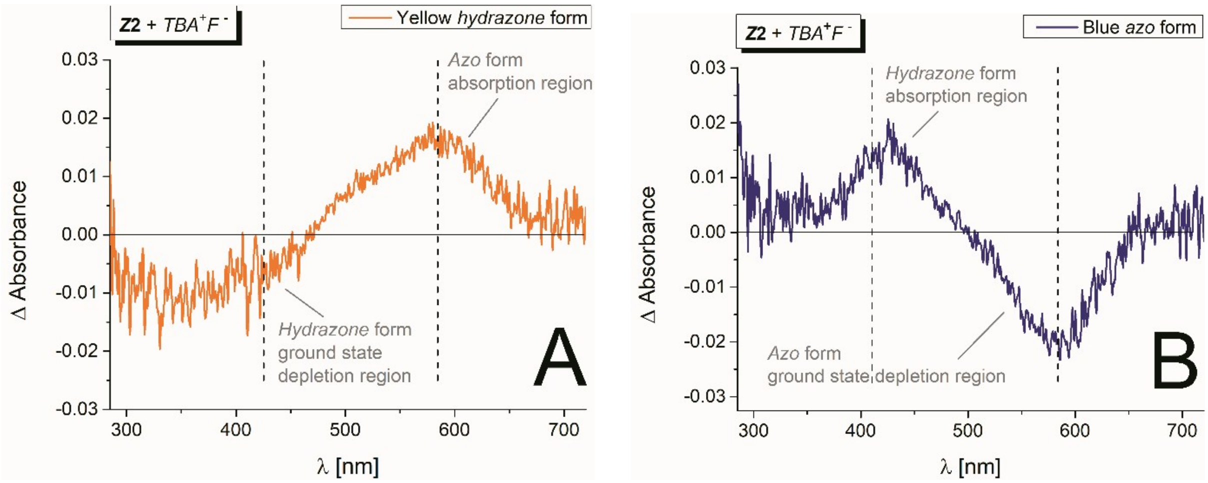

Fig. S11. Transient absorption spectra of the ***Z2***/*TBA*$^+$*F*$^-$ photochromic system in DMSO-d_6 after initial irradiation with light of (A) 590 nm and (B) 470 nm wavelength (λ_{exc} = 355 nm; 1 cm cuvette; T = 298.15 K).

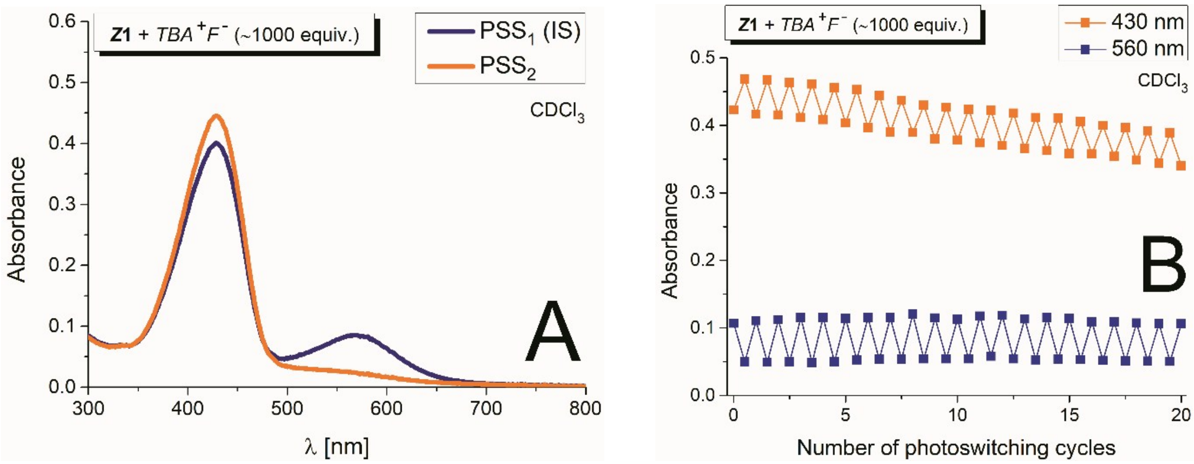

Fig. S12. UV-Vis spectral changes during altered irradiation of the ***Z1***/*TBA*⁺*F*⁻ photochromic system with light of 470 nm and 590 nm wavelength in $CDCl_3$ (0.2 cm cuvette; *T* = 298.15 K; IS – initial state; PSS – photostationary state).

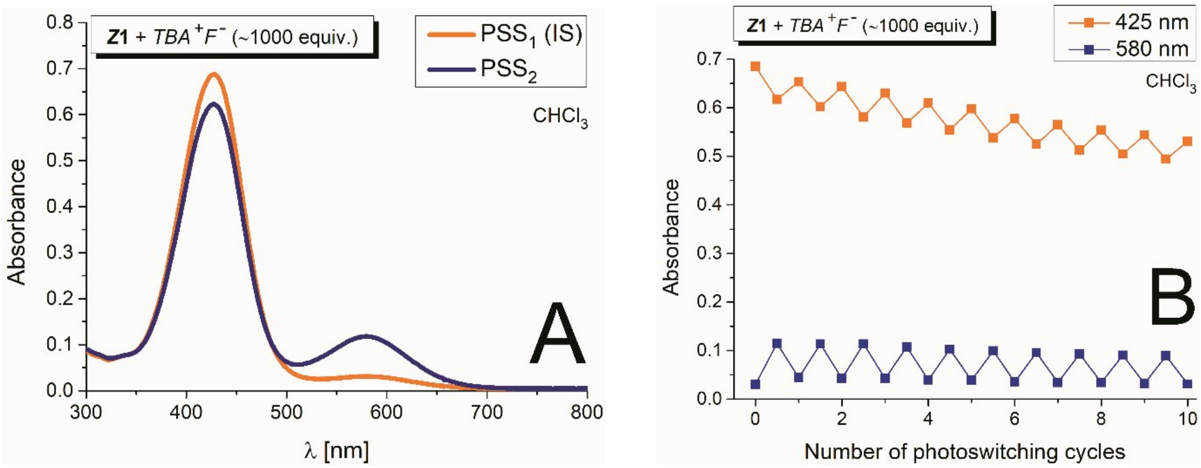

Fig. S13. UV-Vis spectral changes during altered irradiation of the ***Z1***/*TBA*⁺*F*⁻ photochromic system with light of 470 nm and 590 nm wavelength in $CHCl_3$ (0.2 cm cuvette; *T* = 298.15 K; IS – initial state; PSS – photostationary state).

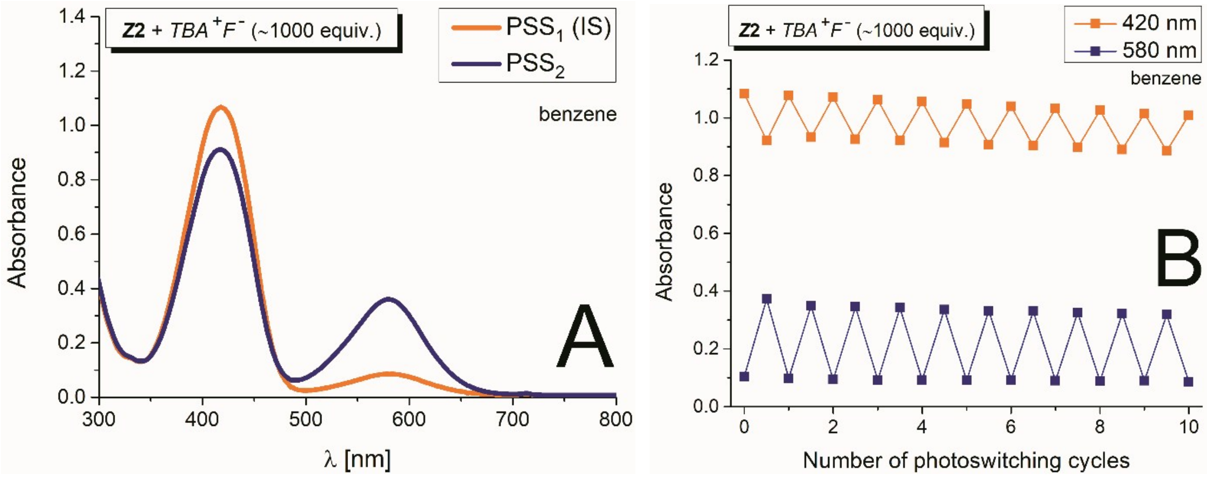

Fig. S14. UV-Vis spectral changes during altered irradiation of the ***Z2***/*TBA*$^+$*F*$^-$ photochromic system with light of 470 nm and 590 nm wavelength in benzene (0.2 cm cuvette; *T* = 298.15 K; IS – initial state; PSS – photostationary state).

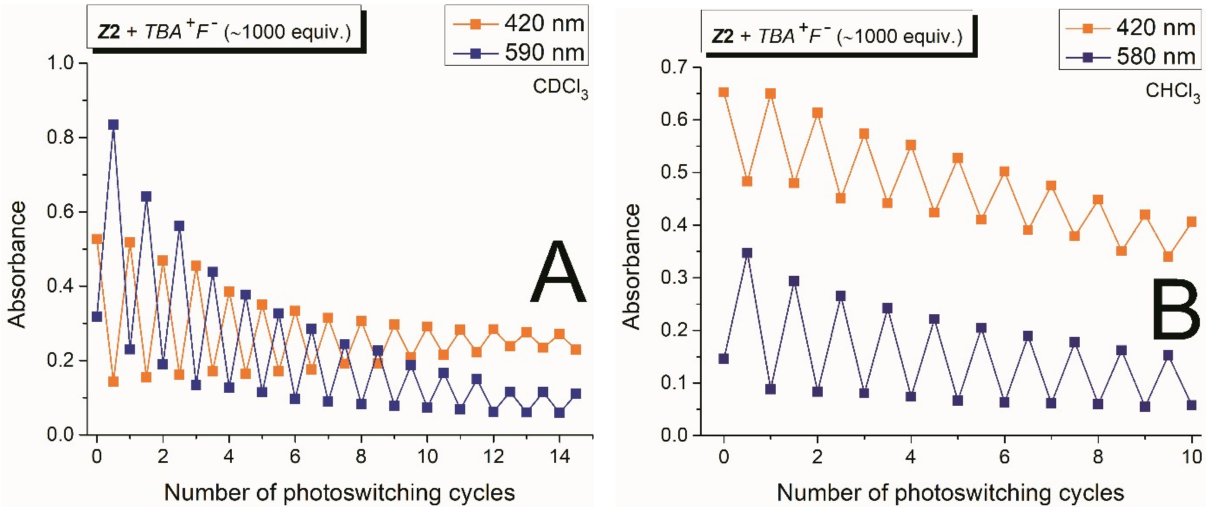

Fig. S15. UV-Vis spectral changes during photoswitching cycles in the ***Z2***/*TBA*$^+$*F*$^-$ photochromic system in (A) $CDCl_3$ and (B) $CHCl_3$ (altered irradiation with light of 470 nm and 590 nm wavelength; 0.2 cm cuvette; *T* = 298.15 K; IS – initial state; PSS – photostationary state).

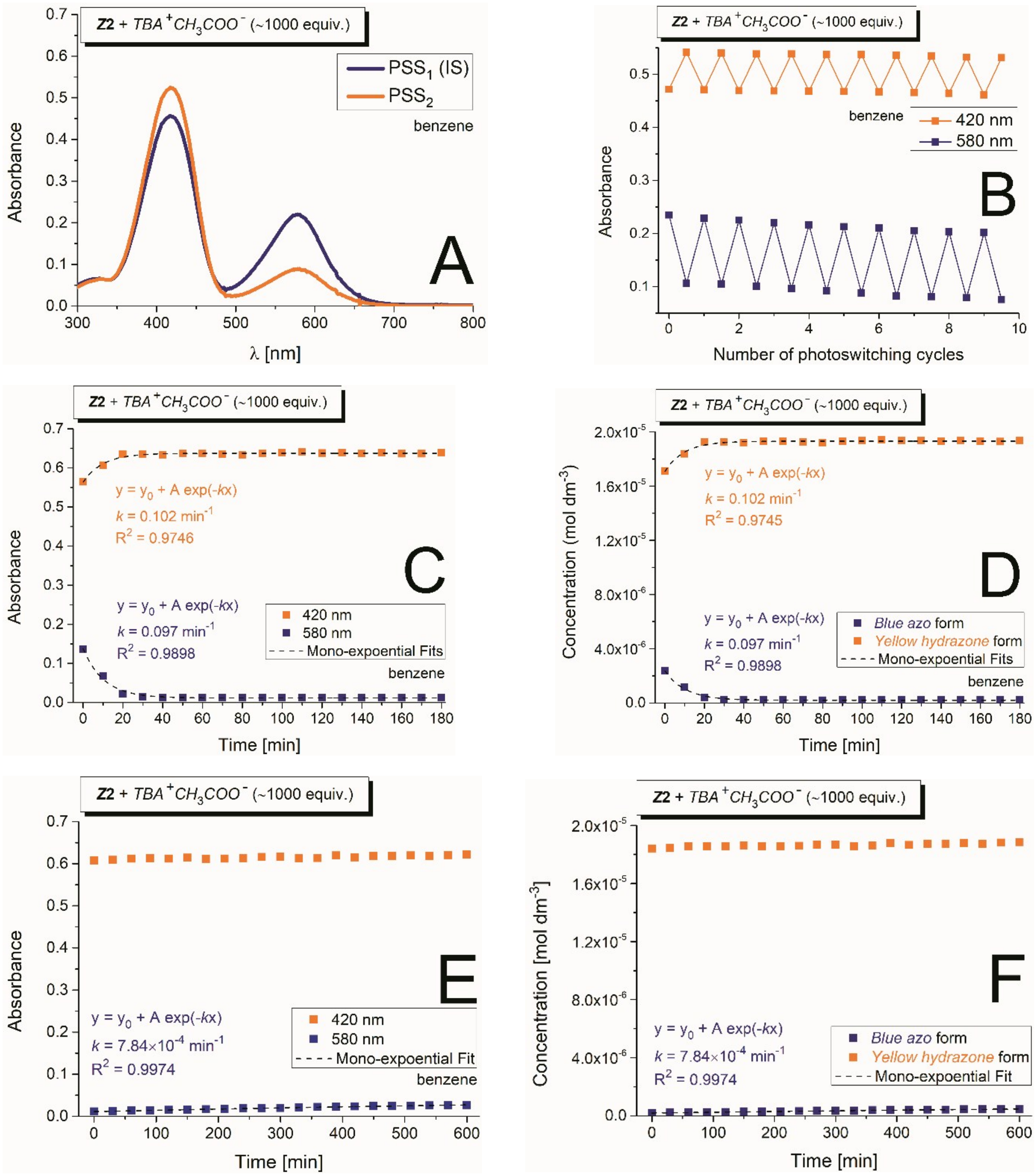

Fig. S16. (A) and (B) UV-Vis spectral changes during photoswitching cycles in the ***Z2***/*TBA*$^+$*CH*$_3$*COO*$^-$ photochromic system in benzene (altered irradiation with light of 470 nm and 590 nm wavelength; 0.2 cm cuvette; *T* = 298.15 K; IS – initial state; PSS – photostationary state). (C) and (D) Time-dependent absorbance (C) and concentration (D) changes of the ***Z2***/*TBA*$^+$*CH*$_3$*COO*$^-$ photochromic system in benzene after initial irradiation with light of 470 nm wavelength (0.2 cm cuvette; *T* = 298.15 K; *k* is the rate constant calculated from mono-exponential fit of absorbance/concentration change of the corresponding form and $t_{1/2}$ is the corresponding thermal reaction half-life). (C) and (D) Time-dependent absorbance (C) and concentration (D) changes of the ***Z2***/*TBA*$^+$*CH*$_3$*COO*$^-$ photochromic system in benzene after initial irradiation with light of 590 nm wavelength.

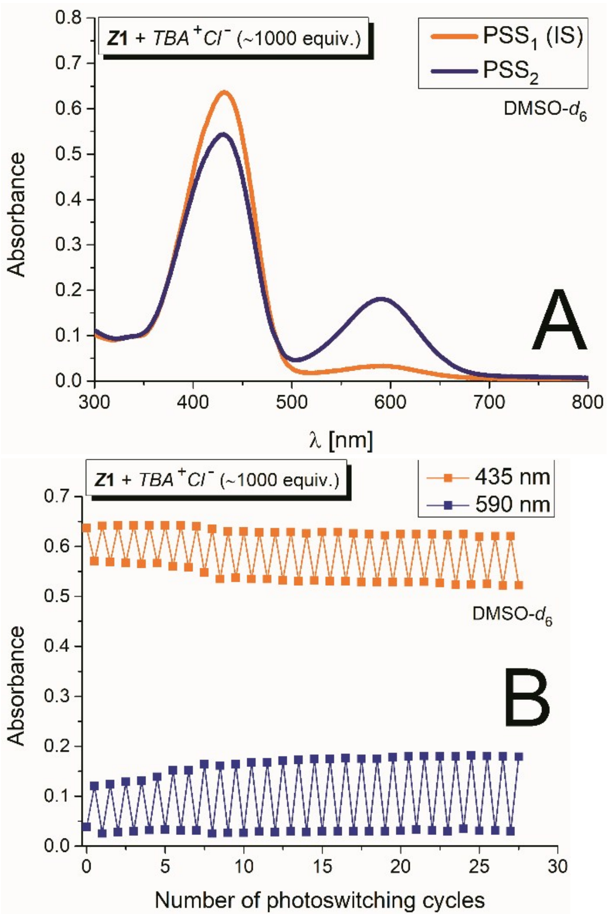

Z1 + TBA+Cl− (~1000 equiv.)
PSS1 (IS)
PSS2
DMSO-d6
A
Absorbance
λ [nm]
Z1 + TBA+Cl− (~1000 equiv.)
435 nm
590 nm
DMSO-d6
B
Absorbance
Number of photoswitching cycles

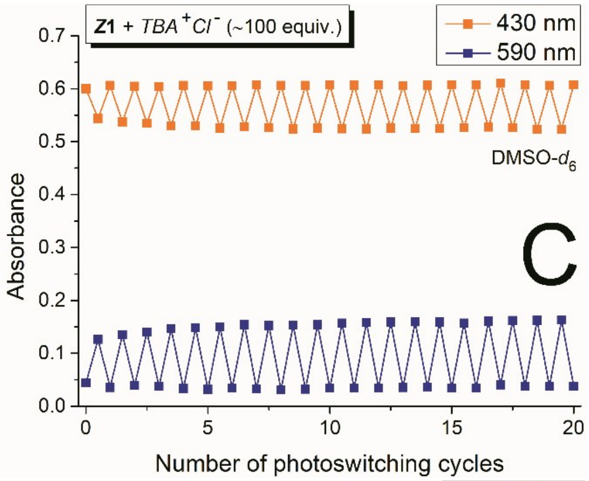

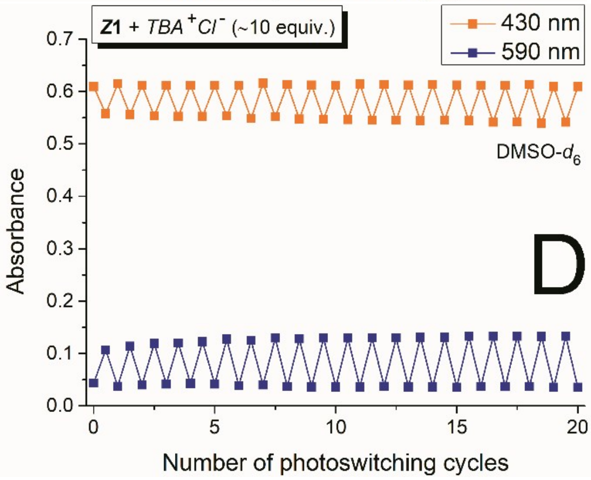

Fig. S17. UV-Vis spectral changes during photoswitching cycles in the ***Z*1**/*TBA*$^+$*Cl*$^-$ photochromic system in DMSO-d_6 (altered irradiation with light of 470 nm and 590 nm wavelength; initial *Z*-isomer concentration: c_{Z1} = 10^{-4} M; 0.2 cm cuvette; T = 298.15 K; IS – initial state; PSS – photostationary state; (A),(B) 1000 equivalents of *TBA*$^+$*Cl*$^-$; (C) 100 equivalents of *TBA*$^+$*Cl*$^-$; (D) 10 equivalents of *TBA*$^+$*Cl*$^-$).

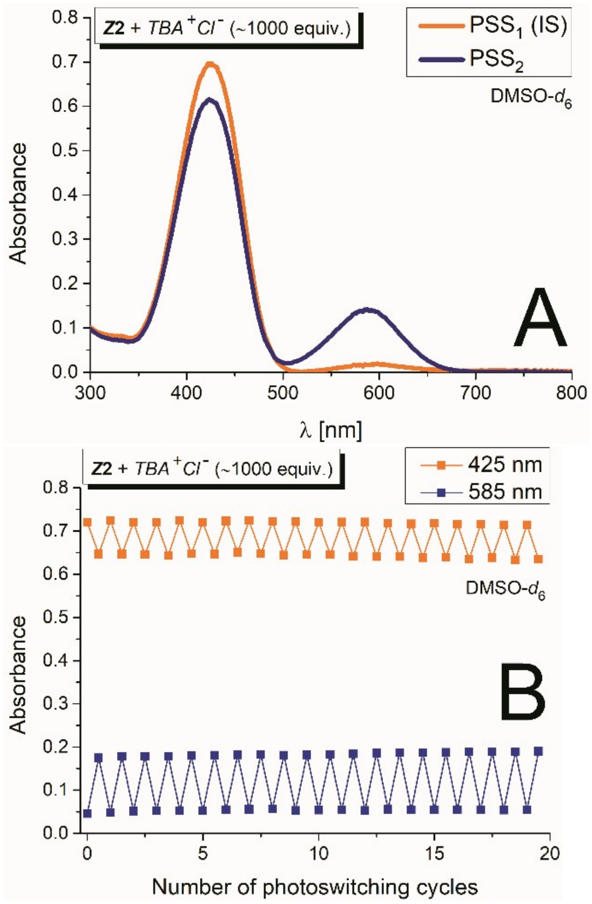

Fig. S18. UV-Vis spectral changes during photoswitching cycles in the ***Z2***/*TBA*$^+$*Cl*$^-$ photochromic system in DMSO-*d*$_6$ (altered irradiation with light of 470 nm and 590 nm wavelength; initial *Z*-isomer concentration: c_{Z2} = 10^{-4} M; 0.2 cm cuvette; *T* = 298.15 K; IS – initial state; PSS – photostationary state; 1000 equivalents of *TBA*$^+$*Cl*$^-$).

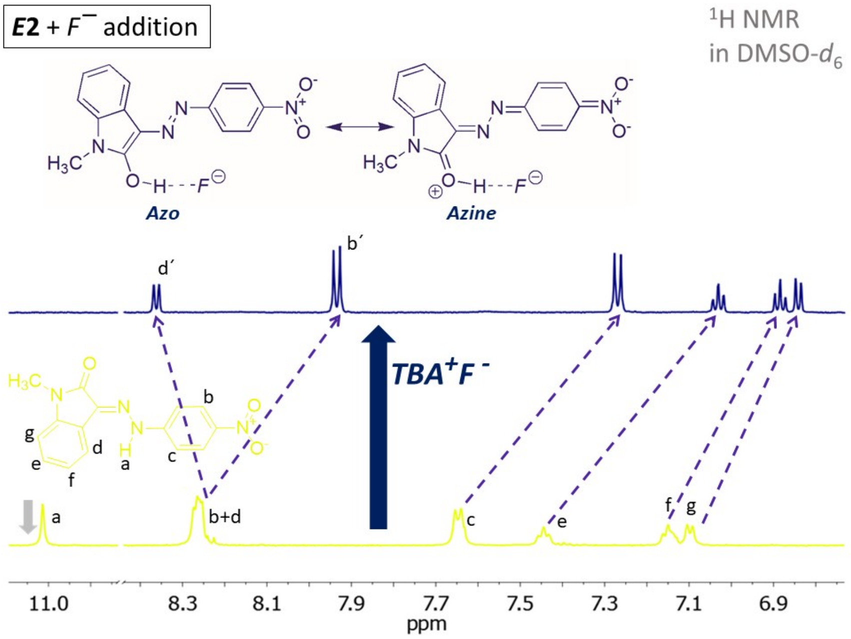

Fig. S19. ¹H NMR spectrum of the ***E2*** in DMSO-d_6 before and after TBA^+F^- addition (1 equivalent of TBA^+F^-; T = 298.15 K).

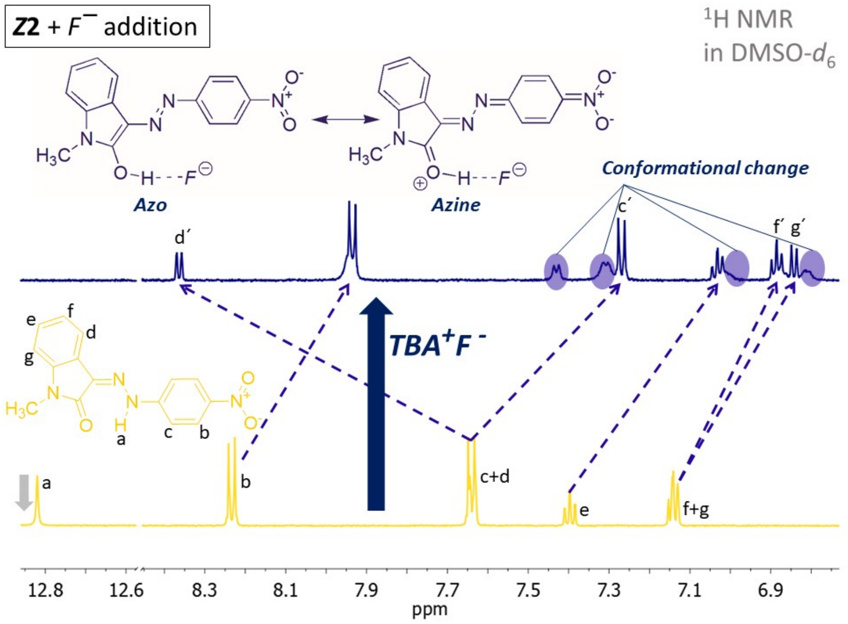

Fig. S20. ¹H NMR spectrum of the ***Z2*** in DMSO-d_6 before and after TBA^+F^- addition (1 equivalent of TBA^+F^-; T = 298.15 K).

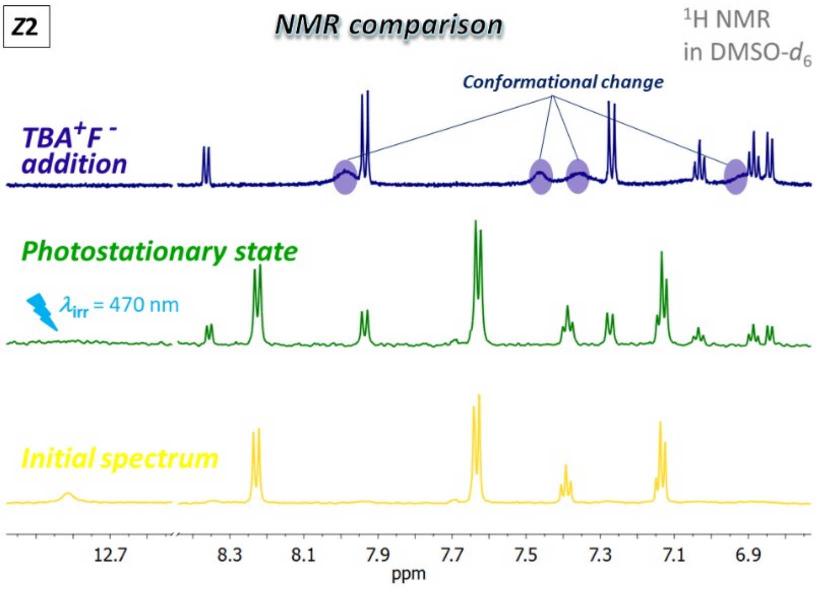

Fig. S21. ^{1}H NMR spectrum of the ***Z2*** in DMSO-d_6 before and after *TBA⁺F⁻* addition (1 equivalent of *TBA⁺F⁻*; *T* = 298.15 K) and in the photostationary state after initial ***Z2*** solution irradiation with light of 470 nm wavelength.

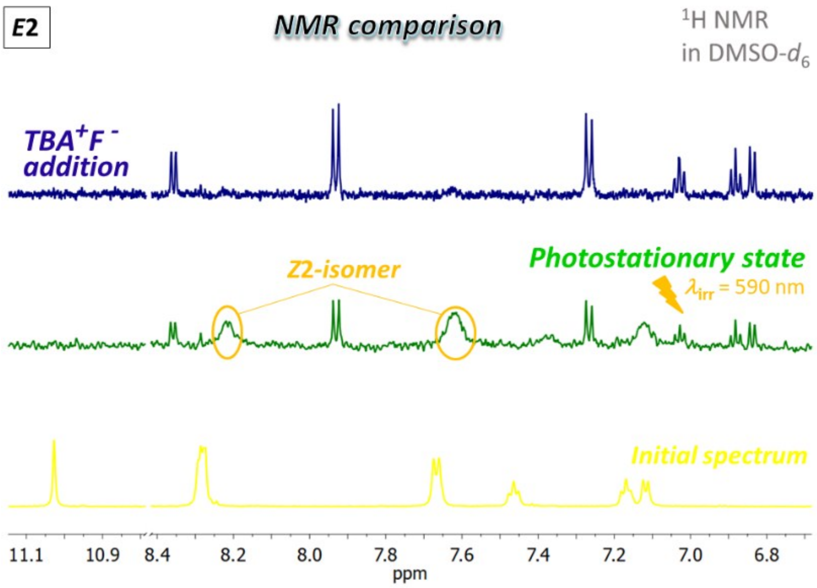

Fig. S22. ^{1}H NMR spectrum of the ***E2*** in DMSO-d_6 before and after *TBA⁺F⁻* addition (1 equivalent of *TBA⁺F⁻*; *T* = 298.15 K) and in the photostationary state after initial ***E2*** solution irradiation with light of 590 nm wavelength.

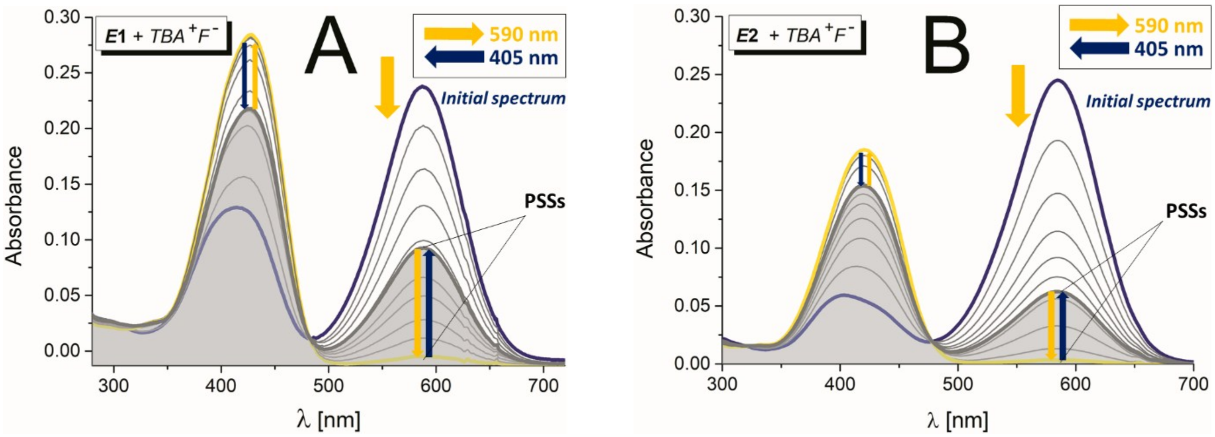

Fig. S23. UV-Vis spectral changes during altered irradiation of the *E* isomer/*TBA*$^{+}$*F*$^{-}$ photochromic system with light of 470 nm and 590 nm wavelength in DMF (0.2 cm cuvette; *T* = 298.15 K; PSSs – photostationary states).

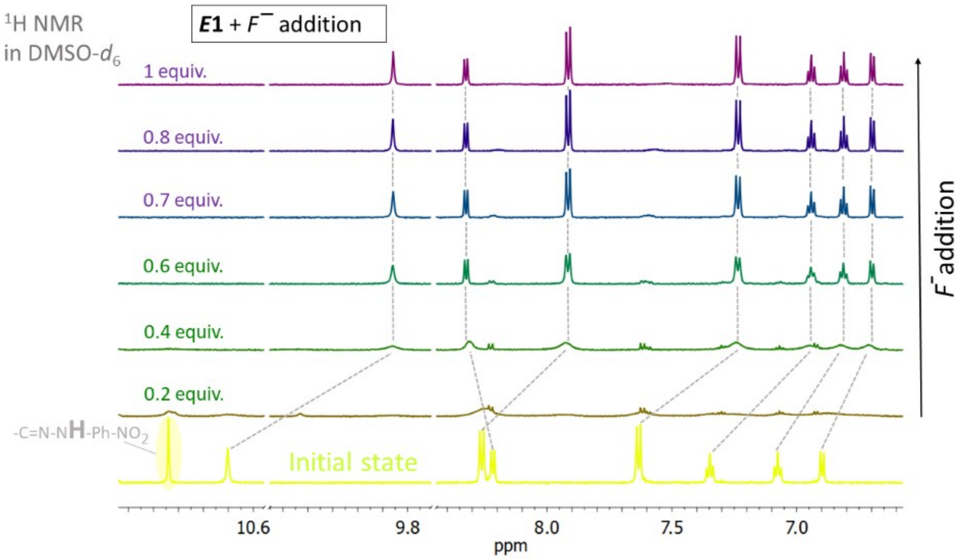

Fig. S24. Evolution of the ^{1}H NMR spectrum of the ***E1*** in DMSO-d_6 during the ***E1*** solution titration with *TBA$^+$F$^-$* (T = 298.15 K).

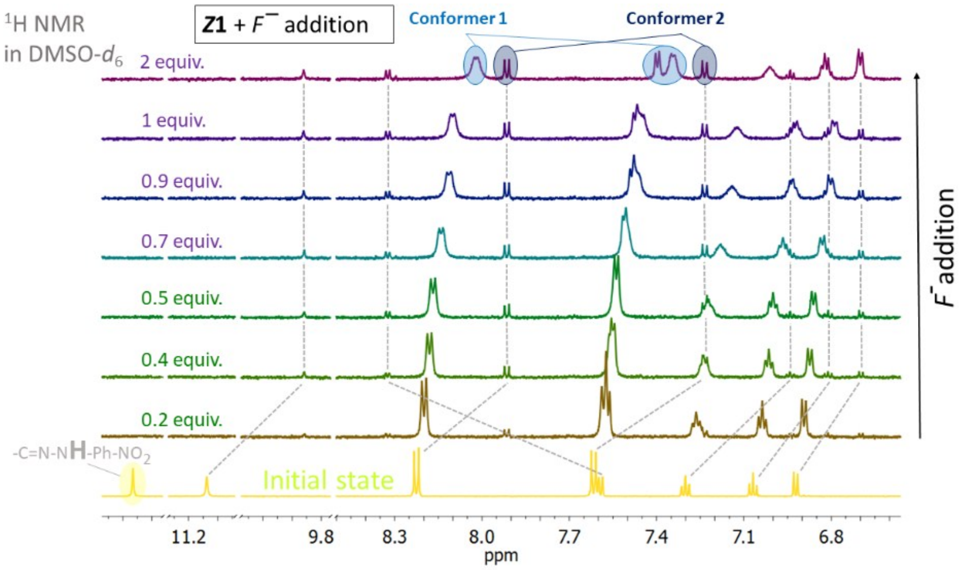

Fig. S25. Evolution of the ^{1}H NMR spectrum of the ***Z1*** in DMSO-d_6 during the ***Z1*** solution titration with *TBA$^+$F$^-$* (T = 298.15 K).

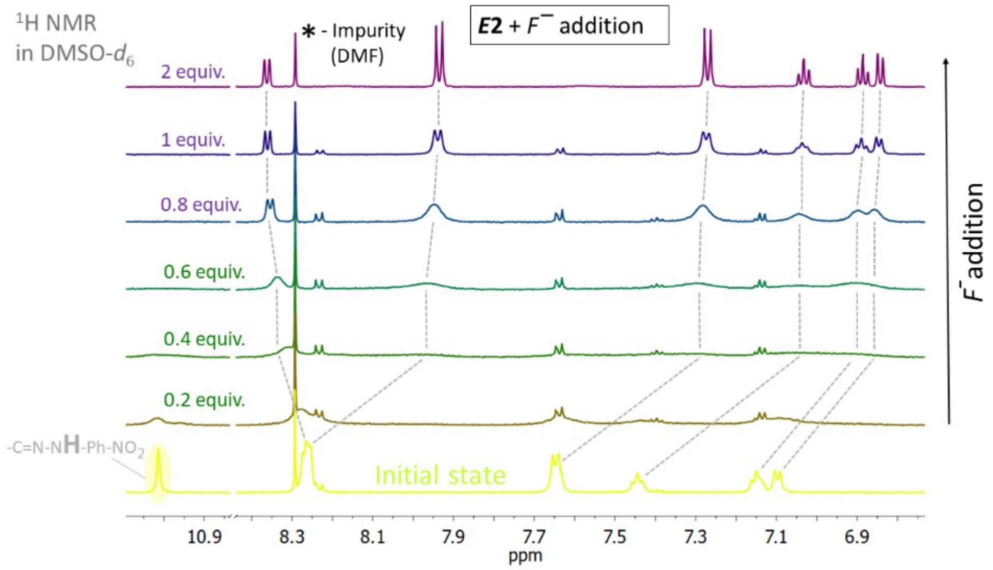

Fig. S26. Evolution of the ^{1}H NMR spectrum of the ***E2*** in DMSO-d_6 during the ***E2*** solution titration with *TBA$^+$F$^-$* (T = 298.15 K).

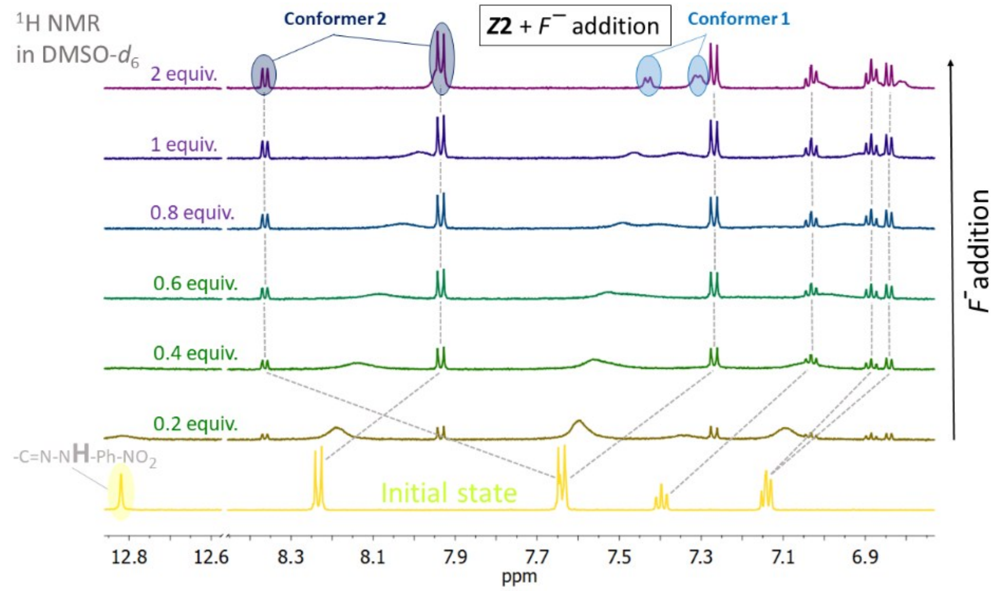

Fig. S27. Evolution of the ^{1}H NMR spectrum of the ***Z2*** in DMSO-d_6 during the ***Z2*** solution titration with *TBA$^+$F$^-$* (T = 298.15 K).

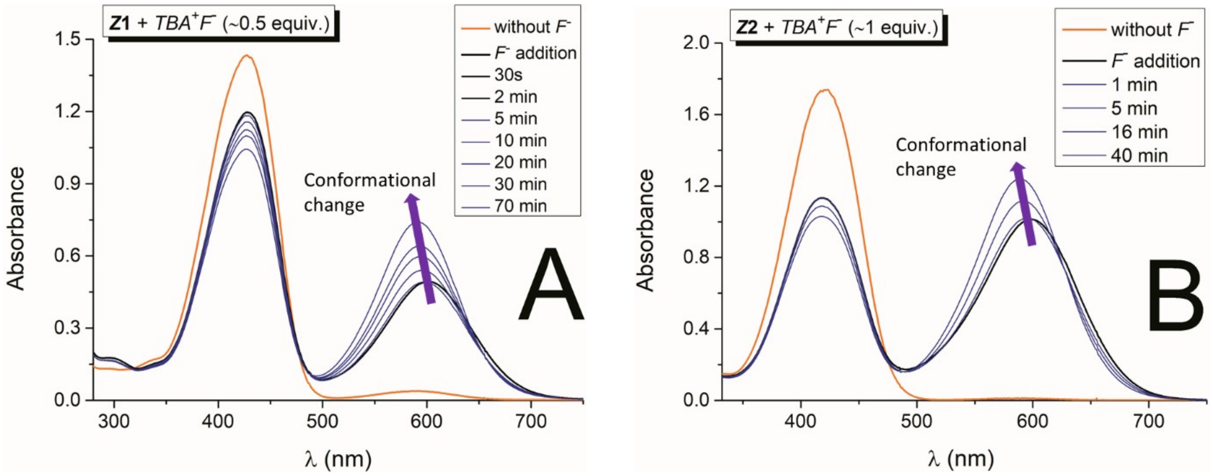

Fig. S28. UV-Vis spectral changes after *TBA*$^+$*F*$^-$ addition to the ***Z*1** and ***Z*2** solution in DMF (1 cm cuvette; *T* = 298.15 K).

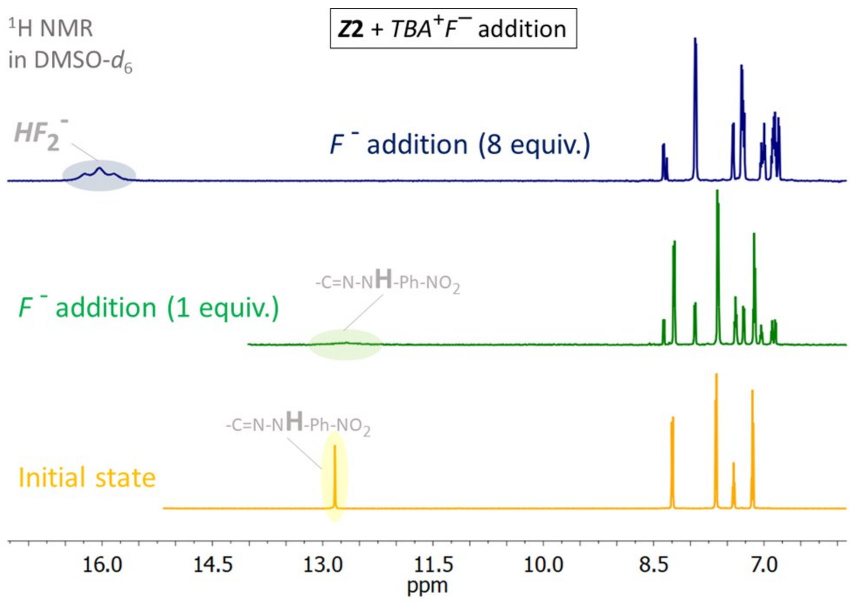

Fig. S29. Evolution of the ^{1}H NMR spectrum of the ***Z2*** in DMSO-d_6 after addition of different equivalents of TBA^+F^- (T = 298.15 K).

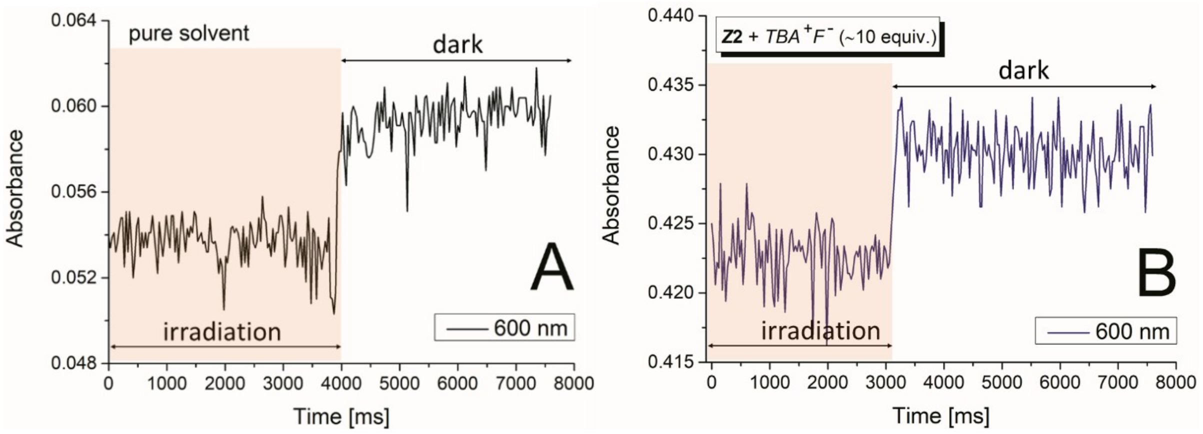

Fig. S30. Ultrafast UV-Vis kinetics measurement of the blue *azo* form during and after 590 nm excitation. (A) blank, (B) sample.

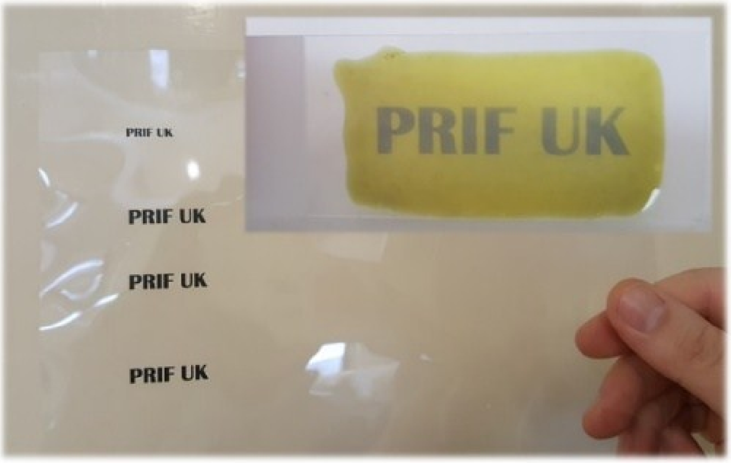

Fig. S31. Logo of our faculty (inset of figure) prepared by irradiation of the blue thin poly (propylene carbonate) film containing ***Z*1**/*TBA*$^+$*F*$^-$ photochromic system with light of 590 nm wavelength through the simple foil photomask.

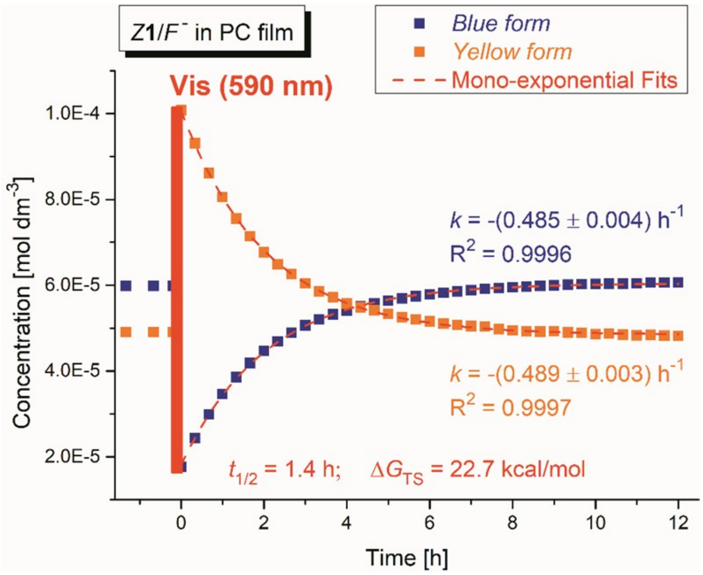

Fig. S32. Concentration changes of the blue and the yellow form of the ***Z*1**/*TBA*$^+$*F*$^-$ photochromic system in poly (propylene carbonate) thin polymer film – back thermal reaction after initial polymer film irradiation with light of 590 nm wavelength (T = 298.15 K; k is the rate constant calculated from mono-exponential fit of concentration change of the corresponding form, $t_{1/2}$ is the half-life of back thermal reaction and ΔG_{TS} is the Gibbs free energy of this reaction).
